# SGLT2 inhibition, circulating metabolites, and atrial fibrillation: a Mendelian randomization study

**DOI:** 10.1186/s12933-023-02019-8

**Published:** 2023-10-17

**Authors:** Jiang Li, Yuefeng Yu, Ying Sun, Bowei Yu, Xiao Tan, Bin Wang, Yingli Lu, Ningjian Wang

**Affiliations:** 1 Institute and Department of Endocrinology and Metabolism, Shanghai Ninth People’s Hospital, Shanghai Jiao Tong University School of Medicine, Shanghai, China; 2 School of Public Health, Zhejiang University, Hangzhou, China; 3 Department of Medical Sciences, Uppsala University, Uppsala, Sweden

**Keywords:** Atrial fibrillation, Sodium-glucose cotransporter 2 inhibition, Circulating metabolites, Mendelian randomization

## Abstract

**Background:**

Sodium-glucose cotransporter 2 (SGLT2) inhibitors have shown promise in reducing the risk of atrial fibrillation (AF). However, the results are controversial and the underlying metabolic mechanism remains unclear. Emerging evidence implied that SGLT2 inhibitors have extra beneficial metabolic effects on circulating metabolites beyond glucose control, which might play a role in reducing the risk of AF. Hence, our study aimed to investigate the effect of circulating metabolites mediating SGLT2 inhibition in AF by Mendelian randomization (MR).

**Methods:**

A two-sample and two-step MR study was conducted to evaluate the association of SGLT2 inhibition with AF and the mediation effects of circulating metabolites linking SGLT2 inhibition with AF. Genetic instruments for SGLT2 inhibition were identified as genetic variants, which were both associated with the expression of *SLC5A2* gene and glycated hemoglobin level (HbA1c). Positive control analysis on type 2 diabetes mellitus (T2DM) was conducted to validate the selection of genetic instruments.

**Results:**

Genetically predicted SGLT2 inhibition (per 1 SD decrement in HbA1c) was associated with reduced risk of T2DM (odds ratio [OR] = 0.63 [95% CI 0.45, 0.88], *P* = 0.006) and AF (0.51 [0.27, 0.97], *P* = 0.039). Among 168 circulating metabolites, two metabolites were both associated with SGLT2 inhibition and AF. The effect of SGLT2 inhibition on AF through the total concentration of lipoprotein particles (0.88 [0.81, 0.96], *P* = 0.004) and the concentration of HDL particles (0.89 [0.82, 0.97], *P* = 0.005), with a mediated proportion of 8.03% (95% CI [1.20%, 14.34%], *P* = 0.010) and 7.59% ([1.09%, 13.34%], *P* = 0.011) of the total effect, respectively.

**Conclusions:**

This study supported the association of SGLT2 inhibition with a reduced risk of AF. The total concentration of lipoprotein particles and particularly the concentration of HDL particles might mediate this association. Further mechanistic and clinical studies research are needed to understand the mediation effects of circulating metabolites especially blood lipids in the association between SGLT2 inhibition and AF.

**Supplementary Information:**

The online version contains supplementary material available at 10.1186/s12933-023-02019-8.

## Introduction

Sodium-glucose cotransporter 2 (SGLT2) inhibitors are a class of oral antidiabetic drugs, which reduce serum glucose concentration by inhibiting the reabsorption of glucose in proximal tubules and promoting urinary glucose excretion. [1] Compelling evidence has shown their benefit in improving cardiovascular, heart failure, and renal outcomes. [2–5] In addition, emerging evidence suggested their potential in decreasing the risk of atrial fibrillation (AF), but the results are controversial and the underlying metabolic mechanism remains unclear. [6–9].

SGLT2 inhibitors were believed to have extra beneficial metabolic effects beyond glycemic control, [10, 11] which might play an important role in improving cardiovascular and renal outcomes. Previous studies have demonstrated that SGLT2 inhibitors have remarkable effects on circulating metabolites, especially amino acids, ketone bodies, and lipids. [12–14] However, the associations between SGLT2 inhibition and circulating metabolites remain unclear, particularly for blood lipids. Some evidence implied that SGLT2 inhibition increased the total cholesterol, low-density lipoprotein (LDL)-cholesterol, and high-density lipoprotein (HDL)-cholesterol levels, [15, 16] whereas others failed to observe a significant change in the serum lipid profile. [17, 18] The discrepancy in these studies might be partially attributed to the limited sample size and the presence of residual confounding.

Mendelian randomization (MR) is a powerful approach that uses genetic variants associated with exposure as instruments to examine the potential causal association between exposure and outcome. MR is less likely to be affected by confounding or reverse causality, [19] as it mimics the randomized controlled trials by randomly assigning genetic variants at the time of conception. [20].

Increasing evidence suggested a correlation between metabolism disorders and the occurrence of AF. [21, 22] In a recent study that comprehensively investigated metabolomic characterization of atrial fibrillation, [23] Lu et al. found that circulating metabolites including amino acids and phospholipids might serve as biomarkers for AF onset and progression. For blood lipids, a systematic review and meta-analysis of large cohort studies found an inverse relationship between total cholesterol, LDL-cholesterol, and HDL-cholesterol levels and AF risk. [24] These findings suggested the potential role of circulating metabolites in the pathogenies of AF.

Therefore, we hypothesized that circulating metabolites might mediate the effect of SGLT2 inhibition on AF. In the present study, we first conducted a two-sample MR to examine the association between SGLT2 inhibition and AF. Second, we performed a two-step MR study to establish the potential metabolic pathway from SGLT2 inhibition to AF through circulating metabolites, particularly blood lipids. We investigated the possible causal effect of SGLT2 inhibition on circulating metabolites, which would provide insights into the metabolic mechanism linking the effect of SGLT2 inhibition with AF.

## Methods

### Study design

**Fig. 1:**
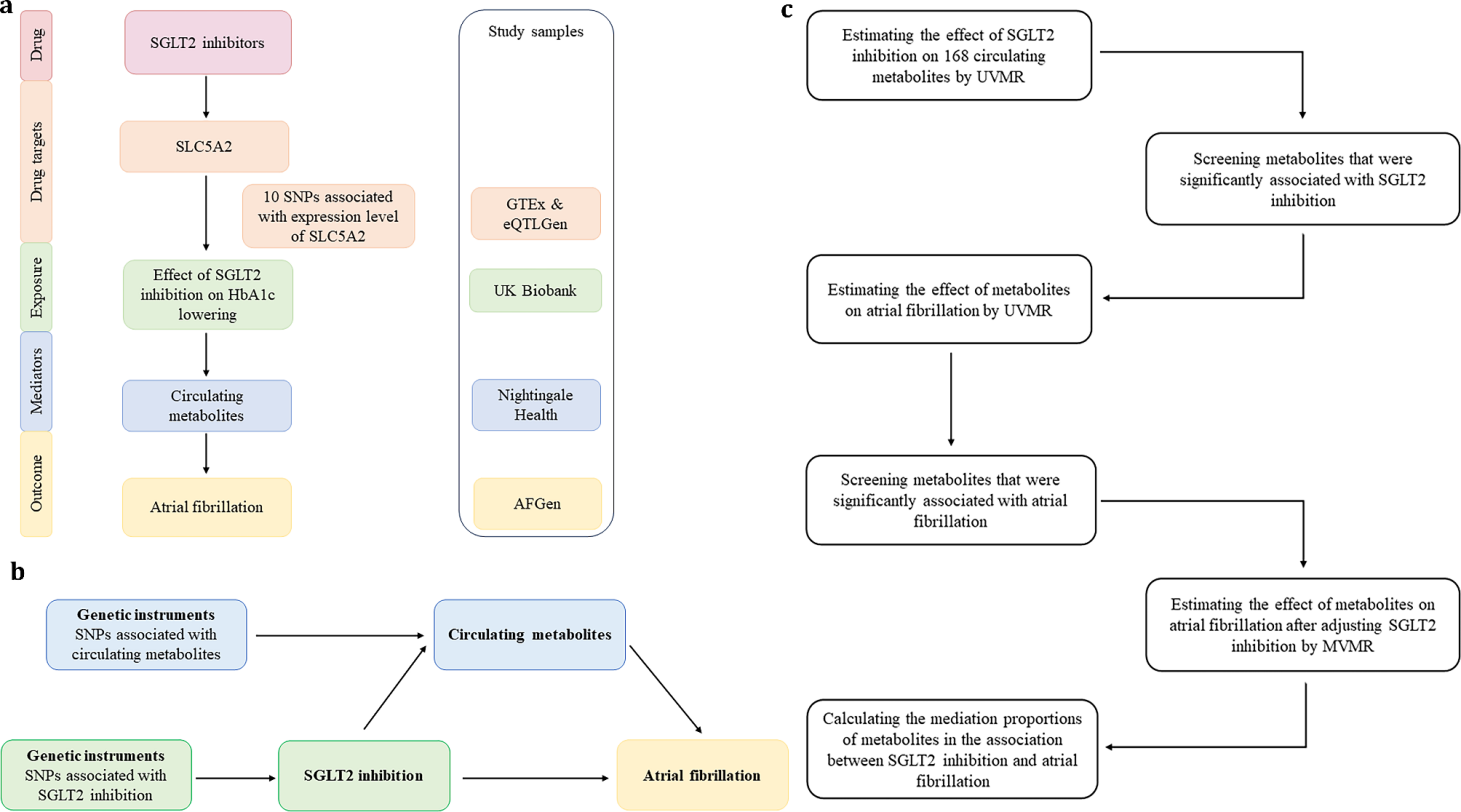
Overview of the study design. (**a**) The flowchart of evaluating the effects of circulating metabolites in mediating the effect of SGLT2 inhibition on atrial fibrillation. (**b**) The framework of the two-step MR. (**c**) The flow diagram of conducting the two-step MR step by step, which involved the selection of circulating metabolites HbA1c, glycated hemoglobin; MVMR, multivariable Mendelian randomization; SNP, single nucleotide polymorphism; UVMR, univariable Mendelian randomization

The current study was conducted using a two-sample MR design (Fig. 1). To ensure the validity of potential causal effects, MR analyses needed to fulfill three core assumptions: [19] (1) genetic variants are robustly associated with the exposure (relevance), (2) genetic variants are independent of confounders (exchangeability), (3) genetic variants influence the outcome only through the exposure (exclusion restriction). This study was reported following the Strengthening the Reporting of Observational Studies in Epidemiology Using Mendelian Randomization (STROBE-MR) guidelines. [25].

### Genetic instruments for SGLT2 inhibition

The identification of genetic variants for SGLT2 inhibition followed four steps (Fig. 1a). First, we used publicly available data from the Genotype-Tissue Expression (GTEx) [26] and eQTLGen Consortium [27] to select genetic variants, which were associated with mRNA expression level of the *SLC5A2* (gene of SGLT2). Second, we estimated the associations of each *SLC5A2* variant with glycated hemoglobin (HbA1c) level, an indicator of glucose-lowering effect, and selected variants that are significantly associated with HbA1c (*P* < 1 × 10^− 4^). The genome-wide association study (GWAS) data of HbA1c was from 344,182 unrelated individuals of European ancestry without diabetes mellitus in the UK Biobank (Supplementary Table 1). Third, colocalization analysis was performed to confirm that *SLC5A*2 and HbA1c share the same causal variant. [28] Evidence for colocalization was defined as a posterior probability > 70% for a shared causal variant. [29] Finally, we clumped those genetic variants to a linkage disequilibrium (LD) of r^2^ < 0.8 within 250 kb using the 1,000 Genomes European reference panel.

### Genetic instruments for circulating metabolites

We systematically searched 249 nuclear magnetic resonance circulating metabolites from 121,000 European ancestry participants generated by Nightingale Health. [30] These metabolic biomarker data comprises 168 biomarker absolute concentrations and 81 biomarker ratios, mainly covering lipids and lipoprotein particles sub-fractions (81%) but also amino acids, cholesterol, esterified cholesterol, free cholesterol, cholines, compounds, fatty acids, glycolysis, ketone bodies, phospholipids, lipoprotein particles size, apolipoproteins, and triglycerides. The absolute concentrations of 168 biomarkers were included for MR analysis. The full GWAS summary statistics of the 168 biomarkers were publicly available through the IEU Open-GWAS Project database with GWAS identifier ‘met-d’ (Supplementary Table 1). [31] We restricted the analyses to genetic variants of each biomarker that were at a genome-wide significant (*P* < 5 × 10^− 8^) and were independent of each other (LD r^2^ < 0.01 within 10,000 kb).

### Study outcomes

We obtained publicly available GWAS summary data for AF from Atrial Fibrillation Genetics (AFGen) Consortium (15,979 cases and 102,776 controls, European ancestry) (Supplementary Table 1). [32] The ascertainment of AF was based on an electrocardiograph recording or outpatient diagnosis. To validate our selection of instruments for SGLT2 inhibition, positive control analysis was performed with type 2 diabetes mellitus (T2DM). The summary data for T2DM were from Diabetes Genetics Replication And Meta-analysis (DIAGRAM) consortium, which accumulated association summary statistics from 122 GWAS in 180,834 T2DM cases and 1,159,055 controls across five ancestry groups. We extracted European ancestry-specific GWAS data (80,154 cases and 853,816 controls) for this study. [33].

### Statistical analyses

#### MR analysis to estimate the effects of SGLT2 inhibition on AF and T2DM

We applied two-sample univariable MR (UVMR) to estimate the effect of SGLT2 inhibition on AF and T2DM. Before MR analysis, we used Mendelian Randomization Pleiotropy RESidual Sum and Outlier (MR-PRESSO) [34] and radial MR [35] to detect and correct possible horizontal pleiotropy and heterogeneity by removing outlying genetic variants. The inverse variant weight (IVW) method was used as the main analysis, which can provide the most precise and powerful estimates when all genetic variants are valid instruments. [36, 37].

### Mediation MR analysis linking SGLT2 inhibition with AF via circulating metabolites

Two-step MR was conducted to estimate the mediation effect of circulating metabolites on the association between SGLT2 inhibition and AF (Fig. 1b and c). First, we estimated the effect of SGLT2 inhibition on 168 circulating metabolites (𝞫1) using UVMR. Second, we evaluated the effect of those metabolites that showed statistically significant associations with SGLT2 inhibition on AF using UVMR. Then, we further screened the metabolites that demonstrated significant associations with AF and performed multivariable MR (MVMR) to evaluate the effect of each metabolite on AF after adjusting for the genetic effect of SGLT2 inhibition (𝞫2). The mediation proportion of each metabolite in the association between SGLT2 inhibition and AF was calculated as the product of 𝞫1and 𝞫2 divided by the total effect of SGLT2 inhibition on AF. The 95% confidence intervals (CIs) of the mediation proportions were calculated through the delta method. [38].

### Sensitivity analysis

In UVMR analysis assessing the effects of SGLT2 inhibition on AF and T2DM, we performed the MR-Egger, MR-PRESSO, weighted median, simple mode, and weighted mode methods as sensitivity analyses. The MR-Egger method examines whether there is directional pleiotropy based on its intercept term, where a value that differs from zero indicates that the presence of directional pleiotropy and the IVW estimate is biased. [39] In addition, the MR-PRESSO method can also determine the presence of directional pleiotropy by detecting possible outliers and recalculating estimates after removing outliers. [34] The weighted median method provides a reliable estimate if at least 50% of the instruments are valid. [40] The simple mode and weighted mode methods provide a reliable estimate when the horizontal pleiotropy in the largest cluster is zero. [41, 42] In UVMR analysis evaluating the effects of SGLT2 inhibition on metabolites and the effects of metabolites on AF, we used MR-Egger and MR-PRESSO methods to validate the robustness of the MV-IVW results. In MVMR, we performed MVMR-Egger methods to validate the robustness.

The strength of the genetic instruments was assessed by F statistics and indicates weak instruments when F statistics < 10. Cochrane’s Q statistics for IVW and the global test for MR-PRESSO were calculated to evaluate the heterogeneity between the instruments.

### Role of the funding source

The funders had no role in the design and conduct of the study; collection, management, analysis, and interpretation of the data; preparation, review, or approval of the manuscript; and decision to submit the manuscript for publication.

All MR analyses were conducted with the package “TwoSampleMR”, “MendelianRandomization”, “MVMR”, “MRPRESSO”, and “RadialMR” in R software (version 4.2.2). To control for multiple testing, a two-sided *P* value that passed the Bonferroni corrected *P* value was defined as statistically significant, but *P* value < 0.05 was considered statistically significant for the effect of SGLT2 inhibition on T2DM and AF since the outcome T2DM was not the primary outcome in the present study.

## Results

### Effect of SGLT2 inhibition on AF and T2DM

In total, 10 independent single nucleotide polymorphisms (SNPs) were selected as genetic instruments for SGLT2 inhibition and the F statistics for all SNPs were greater than 16 (Supplementary Table 2). In MR analysis, we found that SGLT2 inhibition was associated with reduced risk of T2DM (odds ratio [OR] = 0.63 [95% CI 0.45, 0.88], *P* = 0.006) and AF (0.51 [0.27, 0.97], *P* = 0.039), for per 1 SD lowering of HbA1c via SGLT2 inhibition (Table 1). These results were supported by the sensitivity analysis of MR-PRESSO. There was no heterogeneity between instruments for the effect of SGLT2 inhibition on T2DM and AF (Q = 7.010, *P* = 0.536; Q = 1.780, *P* = 0.994), and no horizontal pleiotropy was detected (Egger intercept = 0.026, *P* = 0.115; Egger intercept = 0.012, *P* = 0.680).

**Table 1:** MR estimates of the effect of SGLT2 inhibition on type 2 diabetes mellitus (T2DM) and atrial fibrillation (AF)

Outcome	Method	OR (95% CI)	*P*	Q statistic	*P-*heterogeneity	Egger intercept	*P*-intercept
**T2DM**	IVW	0.63 (0.45, 0.88)	0.006	7.010	0.536		
	MR-Egger	4.11 (0.52, 32.76)	0.224	3.773	0.806	0.026	0.115
	Weighted median	0.67 (0.42, 1.04)	0.077				
	Simple mode	0.58 (0.25, 1.34)	0.238				
	Weighted mode	0.92 (0,45, 1.91)	0.836				
	MR-PRESSO	0.63 (0.46, 0.86)	0.019	9.311	0.552		
**AF**	IVW	0.51 (0.27, 0.97)	0.039	1.780	0.994		
	MR-Egger	1.24 (0.02, 77.44)	0.921	1.596	0.991	0.012	0.680
	Weighted median	0.57 (0.26, 1.22)	0.148				
	Simple mode	0.55 (0.17, 1.76)	0.342				
	Weighted mode	0.59 (0.21, 1.70)	0.355				
	MR-PRESSO	0.51 (0.38, 0.68)	0.001	2.121	0.996		

Odds ratio (OR), 95% confidence interval (CI), and *P* values were calculated for the respective method of MR analysis. The heterogeneity test in the IVW methods was performed using Cochran’s Q statistic and the global test for the MR-PRESSO method. *P* < 0.05 was considered significant

IVW, inverse–variance weighted; *P*-heterogeneity, *P* value for heterogeneity test; *P*-intercept, *P* value for the intercept of MR-Egger regression

### Mediation MR of SGLT2 inhibition, circulating metabolites, and AF

**Fig. 2:**
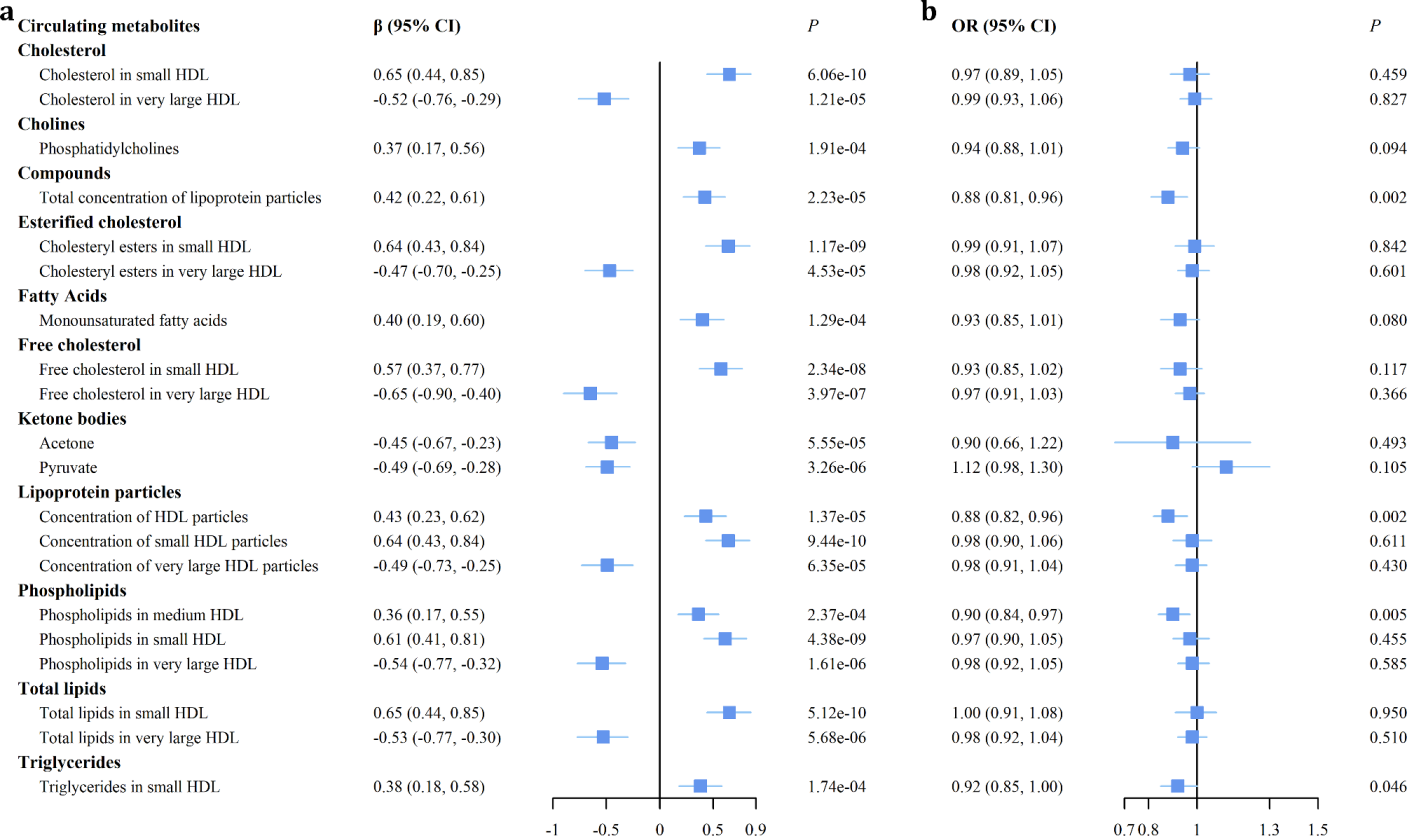
The forest plot of showing the effects of SGLT2 inhibition on circulating metabolites and the effects of metabolites on atrial fibrillation. (**a**) The effects of SGLT2 inhibition on the remaining 20 circulating metabolites selected from 168 metabolites, which were significantly associated with SGLT2 inhibition (Bonferroni-corrected *P* value threshold = 2.98 × 10^− 4^ [0.05/168]). (**b**) The effects of the above 20 metabolites on atrial fibrillation (Bonferroni-corrected *P* value threshold = 0.0025 [0.05/20]) CI, confidence interval; HDL, high-density lipoprotein; OR, odds ratio

We estimated the effect of SGLT2 inhibition on the 168 circulating metabolites and observed 20 metabolites were significantly associated with SGLT2 inhibition (Bonferroni-corrected *P* value threshold = 2.98 × 10^− 4^ [0.05/168]) (Supplementary Table 3, Fig. 2a). For compounds, we observed that SGLT2 inhibition increased the total concentration of lipoprotein particles (𝞫= 0.42 [95% CI 0.22, 0.61], *P* = 2.23 × 10^−5^). For lipoprotein particles, we found that SGLT2 inhibition increased the concentration of HDL particles especially small HDL (0.43 [0.23, 0.62], *P* = 1.37 × 10^−5^ and 0.64 [0.43, 0.84], *P* = 9.44 × 10^−10^, respectively) but decreased the concentration of very large HDL particles (-0.49 [-0.73, -0.25], *P* = 6.35 × 10^−5^).

We further estimated the effect of the 20 circulating metabolites that were significantly associated with SGLT2 inhibition on AF and found that two metabolites were significantly associated with AF (Bonferroni-corrected *P* value threshold = 0.0025 [0.05/20]) (Supplementary Table 4, Fig. 2b). For the total concentration of lipoprotein particles, we observed a negative association with AF (OR 0.88 [95% CI 0.81, 0.96], *P* = 0.002). For the concentration of HDL particles, we also observed a negative association with AF (0.88 [0.82, 0.96], *P* = 0.002). These results were supported by the MR-PRESSO method. There was no evidence of heterogeneity (Q = 58.332, *P* = 0.573; Q = 65.320, *P* = 0.603) and no horizontal pleiotropy (Egger intercept = -0.004, *P* = 0.351; Egger intercept = -0.002, *P* = 0.623). The genetic variants for the 20 metabolites were all strong (F statistics > 23) (Supplementary Data 1–20 are available at 10.6084/m9.figshare.23731140).

**Fig. 3:**
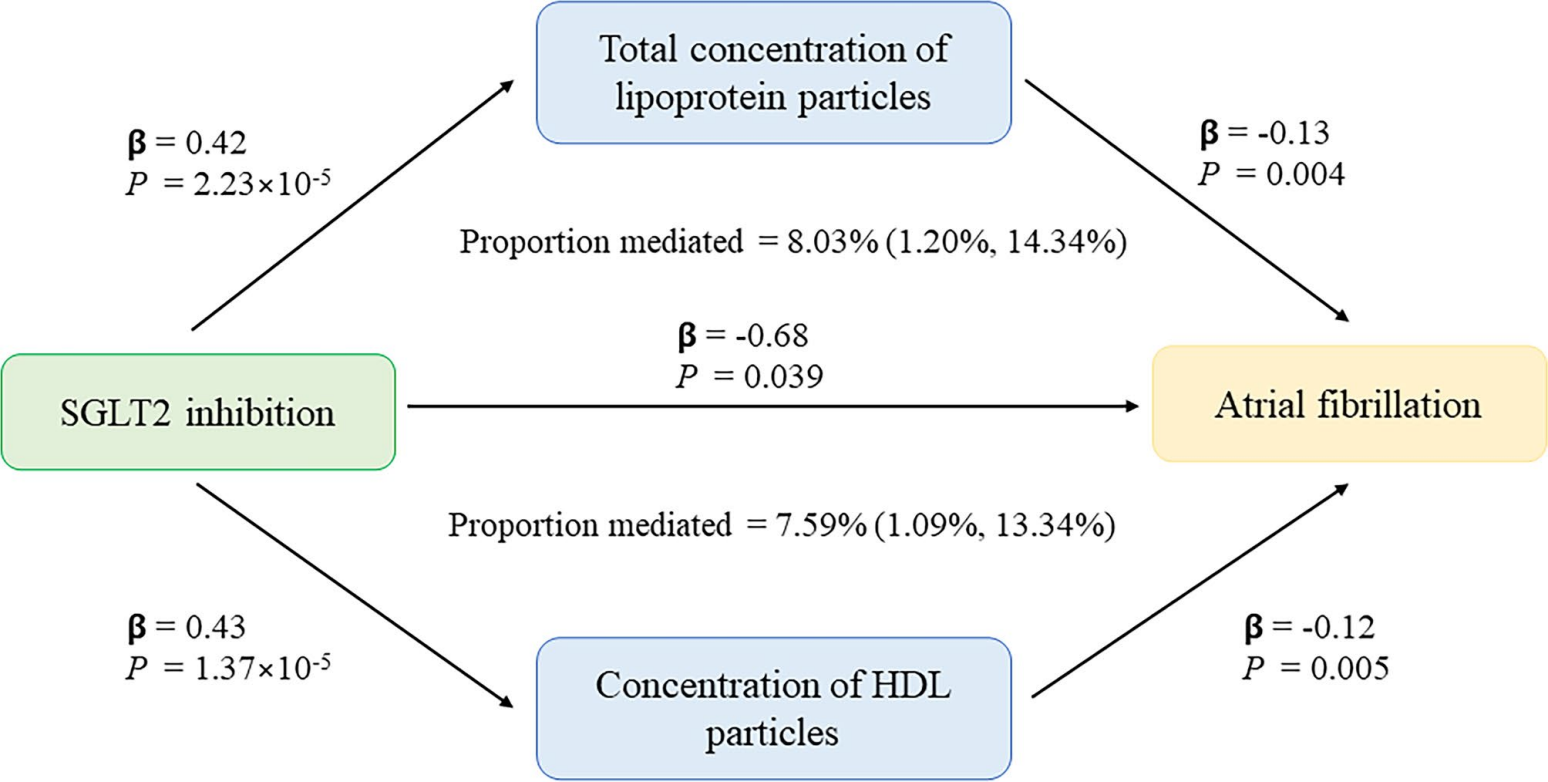
The potential causal evidence summarized from the MR analysis HDL, high-density lipoprotein

We observed an indirect effect of SGLT2 inhibition on AF through the total concentration of lipoprotein particles (OR 0.88 [95% CI 0.81, 0.96], *P* = 0.004) (Bonferroni-corrected *P* value threshold = 0.025 [0.05/2]), with a mediated proportion of 8.03% (95% CI [1.20%, 14.34%], *P* = 0.010) of the total effect (Table 2; Fig. 3). The indirect effect of SGLT2 inhibition on AF through the concentration of HDL particles (OR 0.89 [95% CI 0.82, 0.97], *P* = 0.005) had a mediated proportion of 7.59% (95% CI [1.09%, 13.34%], *P* = 0.011). There was no evidence of heterogeneity and no horizontal pleiotropy in these associations.

**Table 2:** Multivariable MR estimates of the effect of circulating metabolites on atrial fibrillation after adjusting for SGLT2 inhibition

Circulating metabolites	Method	OR (95% CI)	*P*	Q statistic	*P-*heterogeneity	Egger intercept	*P*-intercept
**Compounds**							
Total concentration of lipoprotein particles	MV-IVW	0.88 (0.81, 0.96)	0.004	62.175	0.676		
	MVMR-Egger	0.89 (0.82, 0.98)	0.012	62.283	0.673	0	0.928
**Lipoprotein particles**							
Concentration of HDL particles	MV-IVW	0.89 (0.82, 0.97)	0.005	67.790	0.738		
	MVMR-Egger	1.20 (0.83, 1.73)	0.468	66.669	0.769	-0.004	0.250

Odds ratio (OR), 95% confidence interval (CI), and *P* values were calculated for the respective method of MR analysis. The heterogeneity test in the IVW methods was performed using Cochran’s Q statistic. *P* < 0.025 was considered significant

HDL, high-density lipoprotein. MV-IVW, multivariable inverse–variance weighted; MVMR-Egger, multivariable MR-Egger; *P*-heterogeneity, *P* value for heterogeneity test; *P*-intercept, *P* value for the intercept of MR-Egger regression

## Discussion

### Principal findings

In the present study, we evaluated the associations between genetically predicted SGLT2 inhibition and T2DM and AF. Furthermore, we investigated the mediating role of circulating metabolites in the association between SGLT2 inhibition and AF. Our study indicated that genetic variation in SGLT2 inhibition targets was associated with a lower risk of T2DM (OR 0.63 [95% CI 0.45, 0.88], *P* = 0.006, per 1 SD decrement in HbA1c) and AF (0.51 [0.27, 0.97], *P* = 0.039). The total concentration of lipoprotein particles might mediate 8% of the effect of SGLT2 inhibition on AF, particularly with the concentration of HDL particles mediating 7.6%.

### The association between SGLT2 inhibition and AF

Large clinical trials and meta-analyses have investigated the role of SGLT2 inhibition in incident AF, but the results remain controversial. In the DECLARE-TIMI 58 trial, Dapagliflozin decreased the incidence of AF by 19% (hazard ratio [HR] = 0.81 [95% CI 0.68, 0.95]) in patients with T2DM. [6] However, there was no consistent reduction in incident AF in other randomized clinical trials. [7, 43] In the CANVAS trial, canagliflozin failed to decrease the risk of AF compared with placebo (0.84 [0.64, 1.12]) in T2DM patients. [43] Meta-analyses implied either no effect [8, 44] or possible protective effects [9, 45–49] of SGLT2 against AF. Of note, incident AF was reported as an adverse event rather than a prespecified end point and there were relatively limited AF events in previous randomized clinical trials, which influenced the robustness of the result. [50, 51] Furthermore, previous studies typically focused on patients with diabetes, heart failure, or chronic kidney disease and the protective effect of a certain SGLT2 inhibitor. It remains unclear whether these protective effects could extend to the general population and class of SGLT2 inhibition. [52, 53] Our study provided strong evidence of the protective effect of SGLT2 inhibition on AF in the general population by using a set of robust genetic instruments of SGLT2 as the instrument variables and a large GWAS for AF. Several possible mechanisms have been proposed for the protection against AF by SGLT2 inhibitors, including the modulation of risk factors and off-target actions on cardiomyocytes. [53] The former involves glucose control, weight loss, and reduction in blood pressure. [53] The latter refers to the inhibition effect of SGLT2 inhibitors on sodium-hydrogen exchanger (NHE) activity on cardiomyocytes, which interrupts the increase in intracellular Ca^2+^ concentration and reduces the risk of AF. [53, 54].

### The association between SGLT2 inhibition and circulating metabolites

The effects of SGLT2 inhibitors on blood lipids are conflicting in previous studies. [55, 56] A retrospective study by Calapkulu et al. showed that six months of dapagliflozin treatment in T2DM patients decreased total cholesterol, LDL-cholesterol, and triglyceride (TG) levels. [16] However, a meta-analysis of 48 randomized controlled trials demonstrated that SGLT2 inhibitors significantly increased total cholesterol, LDL-cholesterol, non-HDL-cholesterol, and HDL-cholesterol, and decreased TG levels in patients with T2DM. [15] In addition, some studies indicated no significant change in the lipid profile following SGLT2 administration. [17, 57, 58] A clinical trial on patients with T2DM performed by Bosch et al. revealed that empagliflozin had no significant effect on total cholesterol, HDL-cholesterol, and LDL-cholesterol levels. [58] Inconsistencies in these studies might be related to small sample sizes, retrospective design, potential residual confounding, or short follow-up. [59] More importantly, several studies aimed at investigating the impact of SGLT2 inhibitors on lipoprotein subclasses showed that dapagliflozin treatment for three months did not alter concentrations of LDL-cholesterol, but with a remodeling of LDL particles. [60] Highly atherogenic small dense LDL decreased by 20% and less atherogenic large buoyant LDL-cholesterol increased by 18%. Nevertheless, another randomized trial found that empagliflozin after three months of treatment in patients with T2DM increased total cholesterol, LDL-cholesterol, LDL phospholipids, LDL apolipoprotein B and free fatty acids, but had no effect on LDL particles size. [61] These findings highlighted the importance of distinguishing lipoprotein subclass by size and lipoprotein composition in exploring the effect of SGLT2 inhibition on lipid metabolism.

In our study, we provided insights into the effect of SGLT2 inhibitors on lipid metabolism by using a set of robust genetic variants of SGLT2 as the instruments and the largest circulating metabolites GWAS to date, which mainly covered blood lipids and lipoprotein particles sub-fractions. [30] We observed that SGLT2 inhibition had a significant effect on HDL subclass metabolism. SGLT2 inhibition significantly increased concentration of HDL especially small HDL but decreased levels of very large HDL. For the subclass of lipoprotein composition, we found that SGLT2 inhibition increased cholesterol, esterified cholesterol, free cholesterol, phospholipids, and total lipids levels in small HDL, but decreased these components in very large HDL (Fig. 2a). A randomized clinical trial by Fadini also found that small HDL subfraction levels increased and large HDL subfraction levels decreased after three months of treatment with dapagliflozin, though without statistical significance. [17].

### The mediation effect of circulating metabolites in the association between SGLT2 inhibition and AF

Our findings support that genetically predicted total concentration of lipoprotein particles and concentration of HDL particles were associated with AF, which were consistent with previous findings from one meta-analysis [24] and several cohort studies. [62, 63] The total concentration of lipoprotein particles and concentration of HDL particles might be involved in the association between SGLT2 inhibition and AF. Oxidative stress and chronic inflammation have been proposed to mediate the protective effect of SGLT2 inhibition on AF and previous evidence suggested HDL possesses anti-inflammatory and antioxidant properties. [52, 64, 65] Furthermore, low HDL is an important component in metabolic syndrome, which is associated with an increased risk of AF. [63, 66] Therefore, HDL might mediate this protective effect. Our study provided genetic evidence that HDL might mediate the protective effect of SGLT2 inhibition on AF. Nevertheless, it’s worth noting that the mediation association observed might not be causal and needs to be further validated through experimental studies.

### Strengths and limitations

To our knowledge, this is the first study to use MR analysis to investigate the relationship between SGLT2 inhibition, circulating metabolites, and AF in the general population. Furthermore, we provided genetic evidence on the potential mechanism of SGLT2 inhibition exerting a beneficial effect on AF through the total concentration of lipoprotein particles, primarily HDL. Nevertheless, our study had several limitations. First, genetic variants mimicking SGLT2 inhibition reflect lifelong effects of SGLT2 inhibitors, and the magnitude of these effects might not accurately reflect the magnitude of short-term effects of SGLT2 inhibitors. [67] Hence, MR analysis is more helpful to examine the direction of the potential causal effect rather than quantifying its magnitude. However, the expected direction of effects can inform potential efficacy, which can be further explored in experiments and clinical trials. Second, there are overlapped samples of GWAS for SGLT2 inhibition and the circulating metabolites, the GWAS for Nightingale Health metabolites was conducted in around 24.2% of the UK Biobank. In the case of the weak instruments, sample overlap between exposure and outcome could bias the potential causal effect estimate by introducing associations between instruments and confounders. [68] However, the genetic variants for SGLT2 inhibition in this study were strongly associated with exposure indicated by high F-statistics (all > 16), which implied that our results were unlikely biased by weak instruments. Third, the lack of large public GWAS data for lipids and lipids subfractions like metabolic profiling by Nightingale Health limited further validation of our findings. [69] Finally, as this study was performed using data from individuals of European ancestry, the generalization of these results to other populations warrants further investigation.

## Conclusions

In conclusion, this study supported the association between genetically predicted SGLT2 inhibition, circulating metabolites, and AF. Specifically, the total concentration of lipoprotein particles, especially HDL particle concentration, mediated the protective effect of SGLT2 inhibition on AF. These findings provided genetic evidence for the mechanisms of SGLT2 inhibition in reducing AF risk and might inform future mechanistic and clinical studies.

### Electronic supplementary material

Below is the link to the electronic supplementary material.

Supplementary Material 1

## Data Availability

The GWAS Summary statistics used in this study were publicly accessed from the IEU OpenGWAS project (https://gwas.mrcieu.ac.uk/), the GTEx Portal (https://www.gtexportal.org/), the eQTLGen Consortium (https://eqtlgen.org/) and the DIAGRAM consortium (https://diagram-consortium.org/).

## Abbreviations

AF: atrial fibrillation
GWAS: genome-wide association study
HbA1c: glycated hemoglobin
HDL: high-density lipoprotein
IVW: inverse variant weight
LDL: low-density lipoprotein
MR: Mendelian randomization
MVMR: multivariable Mendelian randomization
SGLT2: Sodium-glucose cotransporter 2 T2DM: type 2 diabetes mellitus
UVMR: univariable Mendelian randomization
