# Supplementary material for: SGLT2 inhibition, circulating metabolites, and atrial fibrillation: a Mendelian randomization study
Source: Cardiovasc Diabetol. 2023 Oct 17;22:278. doi: 10.1186/s12933-023-02019-8 (PMC10583416; doi:10.1186/s12933-023-02019-8)
Supplement: Supplementary file 1 — Supplementary Material 1 [file 12933_2023_2019_MOESM1_ESM.docx]

**SGLT2 inhibition, circulating metabolites, and atrial** **fibrillation: a Mendelian randomization study**

**Supplementary Tables**

Supplementary Table 1. Detailed information for genome-wide association study (GWAS) statistics used in the present study

Supplementary Table 2. Instrumental variables for SGLT2 inhibition

Supplementary Table 3. MR estimates of the effect of SGLT2 inhibition on circulating metabolites

Supplementary Table 4. MR estimates of the effect of circulating metabolites on atrial fibrillation

| **Supplementary Table 1. Detailed information for genome-wide association study (GWAS) statistics used in the present study** | | | | | |
| --- | --- | --- | --- | --- | --- |
| Phenotype | Consortium | Sample size | Ancestry | Year | IEU OpenGWAS ID or study reference |
| SGLT2 inhibition | UK Biobank | 344,182 | European | 2018 | ukb-d-30750_irnt |
| Type 2 diabetes mellitus | DIAGRAM | 933,970 | European | 2022 | Nat Genet. 2022; 54(5):560-572 |
| Atrilal fibrillation | AFGen | 118,755 | European | 2017 | ebi-a-GCST004296, Nat Genet. 2017; 49(6):946-952 |
| **Circulating metabolities** |  |  |  |  |  |
| **Amino acids** |  |  |  |  |  |
| Alanine | Nightingale Health | 115,074 | European | 2020 | met-d-Ala |
| Glutamine | Nightingale Health | 114,750 | European | 2020 | met-d-Gln |
| Glycine | Nightingale Health | 114,972 | European | 2020 | met-d-Gly |
| Histidine | Nightingale Health | 114,895 | European | 2020 | met-d-His |
| Isoleucine | Nightingale Health | 115,075 | European | 2020 | met-d-Ile |
| Leucine | Nightingale Health | 115,074 | European | 2020 | met-d-Leu |
| Phenylalanine | Nightingale Health | 115,025 | European | 2020 | met-d-Phe |
| Tyrosine | Nightingale Health | 114,911 | European | 2020 | met-d-Tyr |
| Valine | Nightingale Health | 115,048 | European | 2020 | met-d-Val |
| **Cholesterol** |  |  |  |  |  |
| Cholesterol in IDL | Nightingale Health | 115,078 | European | 2020 | met-d-IDL_C |
| Cholesterol in chylomicrons and extremely large VLDL | Nightingale Health | 115,078 | European | 2020 | met-d-XXL_VLDL_C |
| Cholesterol in large HDL | Nightingale Health | 115,078 | European | 2020 | met-d-L_HDL_C |
| Cholesterol in large LDL | Nightingale Health | 115,078 | European | 2020 | met-d-L_LDL_C |
| Cholesterol in large VLDL | Nightingale Health | 115,078 | European | 2020 | met-d-L_VLDL_C |
| Cholesterol in medium HDL | Nightingale Health | 115,078 | European | 2020 | met-d-M_HDL_C |
| Cholesterol in medium LDL | Nightingale Health | 115,078 | European | 2020 | met-d-M_LDL_C |
| Cholesterol in medium VLDL | Nightingale Health | 115,078 | European | 2020 | met-d-M_VLDL_C |
| Cholesterol in small HDL | Nightingale Health | 115,078 | European | 2020 | [met-d-S_HDL_C](https://gwas.mrcieu.ac.uk/datasets/met-d-S_HDL_C/) |
| Cholesterol in small LDL | Nightingale Health | 115,078 | European | 2020 | [met-d-S_LDL_C](https://gwas.mrcieu.ac.uk/datasets/met-d-S_LDL_C/) |
| Cholesterol in small VLDL | Nightingale Health | 115,078 | European | 2020 | [met-d-S_VLDL_C](https://gwas.mrcieu.ac.uk/datasets/met-d-S_VLDL_C/) |
| Cholesterol in very large HDL | Nightingale Health | 115,078 | European | 2020 | [met-d-XL_HDL_C](https://gwas.mrcieu.ac.uk/datasets/met-d-XL_HDL_C/) |
| Cholesterol in very large VLDL | Nightingale Health | 115,078 | European | 2020 | [met-d-XL_VLDL_C](https://gwas.mrcieu.ac.uk/datasets/met-d-XL_VLDL_C/) |
| Cholesterol in very small VLDL | Nightingale Health | 115,078 | European | 2020 | [met-d-XS_VLDL_C](https://gwas.mrcieu.ac.uk/datasets/met-d-XS_VLDL_C/) |
| **Cholines** |  |  |  |  |  |
| Phosphatidylcholines | Nightingale Health | 114,999 | European | 2020 | [met-d-Phosphatidylc](https://gwas.mrcieu.ac.uk/datasets/met-d-Phosphatidylc/) |
| Phosphoglycerides | Nightingale Health | 114,999 | European | 2020 | [met-d-Phosphoglyc](https://gwas.mrcieu.ac.uk/datasets/met-d-Phosphoglyc/) |
| Sphingomyelins | Nightingale Health | 114,999 | European | 2020 | [met-d-Sphingomyelins](https://gwas.mrcieu.ac.uk/datasets/met-d-Sphingomyelins/) |
| Total cholines | Nightingale Health | 114,999 | European | 2020 | [met-d-Cholines](https://gwas.mrcieu.ac.uk/datasets/met-d-Cholines/) |
| **Compounds** |  |  |  |  |  |
| Clinical LDL cholesterol | Nightingale Health | 115,078 | European | 2020 | [met-d-Clinical_LDL_C](https://gwas.mrcieu.ac.uk/datasets/met-d-Clinical_LDL_C/) |
| HDL cholesterol | Nightingale Health | 115,078 | European | 2020 | [met-d-HDL_C](https://gwas.mrcieu.ac.uk/datasets/met-d-HDL_C/) |
| LDL cholesterol | Nightingale Health | 115,078 | European | 2020 | met-d-LDL_C |
| Remnant cholesterol (non-HDL, non-LDL -cholesterol) | Nightingale Health | 115,078 | European | 2020 | [met-d-Remnant_C](https://gwas.mrcieu.ac.uk/datasets/met-d-Remnant_C/) |
| Total cholesterol | Nightingale Health | 115,078 | European | 2020 | met-d-Total_C |
| Total cholesterol minus HDL-C | Nightingale Health | 115,078 | European | 2020 | [met-d-non_HDL_C](https://gwas.mrcieu.ac.uk/datasets/met-d-non_HDL_C/) |
| Total concentration of branched-chain amino acids (leucine + isoleu | Nightingale Health | 115,047 | European | 2020 | [met-d-Total_BCAA](https://gwas.mrcieu.ac.uk/datasets/met-d-Total_BCAA/) |
| Total concentration of lipoprotein particles | Nightingale Health | 115,078 | European | 2020 | [met-d-Total_P](https://gwas.mrcieu.ac.uk/datasets/met-d-Total_P/) |
| Total esterified cholesterol | Nightingale Health | 115,078 | European | 2020 | [met-d-Total_CE](https://gwas.mrcieu.ac.uk/datasets/met-d-Total_CE/) |
| Total free cholesterol | Nightingale Health | 115,078 | European | 2020 | [met-d-Total_FC](https://gwas.mrcieu.ac.uk/datasets/met-d-Total_FC/) |
| Total phospholipids in lipoprotein particles | Nightingale Health | 115,078 | European | 2020 | [met-d-Total_PL](https://gwas.mrcieu.ac.uk/datasets/met-d-Total_PL/) |
| Total triglycerides | Nightingale Health | 115,078 | European | 2020 | [met-d-Total_TG](https://gwas.mrcieu.ac.uk/datasets/met-d-Total_TG/) |
| VLDL cholesterol | Nightingale Health | 115,078 | European | 2020 | [met-d-VLDL_C](https://gwas.mrcieu.ac.uk/datasets/met-d-VLDL_C/) |
| **Esterified cholesterol** |  |  |  |  |  |
| Cholesteryl esters in HDL | Nightingale Health | 115,078 | European | 2020 | met-d-HDL_CE |
| Cholesteryl esters in IDL | Nightingale Health | 115,078 | European | 2020 | [met-d-IDL_CE](https://gwas.mrcieu.ac.uk/datasets/met-d-IDL_CE/) |
| Cholesteryl esters in LDL | Nightingale Health | 115,078 | European | 2020 | [met-d-LDL_CE](https://gwas.mrcieu.ac.uk/datasets/met-d-LDL_CE/) |
| Cholesteryl esters in VLDL | Nightingale Health | 115,078 | European | 2020 | [met-d-VLDL_CE](https://gwas.mrcieu.ac.uk/datasets/met-d-VLDL_CE/) |
| Cholesteryl esters in chylomicrons and extremely large VLDL | Nightingale Health | 115,078 | European | 2020 | [met-d-XXL_VLDL_CE](https://gwas.mrcieu.ac.uk/datasets/met-d-XXL_VLDL_CE/) |
| Cholesteryl esters in large HDL | Nightingale Health | 115,078 | European | 2020 | [met-d-L_HDL_CE](https://gwas.mrcieu.ac.uk/datasets/met-d-L_HDL_CE/) |
| Cholesteryl esters in large LDL | Nightingale Health | 115,078 | European | 2020 | [met-d-L_LDL_CE](https://gwas.mrcieu.ac.uk/datasets/met-d-L_LDL_CE/) |
| Cholesteryl esters in large VLDL | Nightingale Health | 115,078 | European | 2020 | [met-d-L_VLDL_CE](https://gwas.mrcieu.ac.uk/datasets/met-d-L_VLDL_CE/) |
| Cholesteryl esters in medium HDL | Nightingale Health | 115,078 | European | 2020 | [met-d-M_HDL_CE](https://gwas.mrcieu.ac.uk/datasets/met-d-M_HDL_CE/) |
| Cholesteryl esters in medium LDL | Nightingale Health | 115,078 | European | 2020 | [met-d-M_LDL_CE](https://gwas.mrcieu.ac.uk/datasets/met-d-M_LDL_CE/) |
| Cholesteryl esters in medium VLDL | Nightingale Health | 115,078 | European | 2020 | [met-d-M_VLDL_CE](https://gwas.mrcieu.ac.uk/datasets/met-d-M_VLDL_CE/) |
| Cholesteryl esters in small HDL | Nightingale Health | 115,078 | European | 2020 | [met-d-S_HDL_CE](https://gwas.mrcieu.ac.uk/datasets/met-d-S_HDL_CE/) |
| Cholesteryl esters in small LDL | Nightingale Health | 115,078 | European | 2020 | [met-d-S_LDL_CE](https://gwas.mrcieu.ac.uk/datasets/met-d-S_LDL_CE/) |
| Cholesteryl esters in small VLDL | Nightingale Health | 115,078 | European | 2020 | [met-d-S_VLDL_CE](https://gwas.mrcieu.ac.uk/datasets/met-d-S_VLDL_CE/) |
| Cholesteryl esters in very large HDL | Nightingale Health | 115,078 | European | 2020 | [met-d-XL_HDL_CE](https://gwas.mrcieu.ac.uk/datasets/met-d-XL_HDL_CE/) |
| Cholesteryl esters in very large VLDL | Nightingale Health | 115,078 | European | 2020 | [met-d-XL_VLDL_CE](https://gwas.mrcieu.ac.uk/datasets/met-d-XL_VLDL_CE/) |
| Cholesteryl esters in very small VLDL | Nightingale Health | 115,078 | European | 2020 | [met-d-XS_VLDL_CE](https://gwas.mrcieu.ac.uk/datasets/met-d-XS_VLDL_CE/) |
| **Fatty Acids** |  |  |  |  |  |
| Total fatty acids | Nightingale Health | 114,999 | European | 2020 | met-d-Total_FA |
| Degree of unsaturation | Nightingale Health | 114,999 | European | 2020 | [met-d-Unsaturation](https://gwas.mrcieu.ac.uk/datasets/met-d-Unsaturation/) |
| Docosahexaenoic acid | Nightingale Health | 114,999 | European | 2020 | [met-d-DHA](https://gwas.mrcieu.ac.uk/datasets/met-d-DHA/) |
| Linoleic acid | Nightingale Health | 114,999 | European | 2020 | met-d-LA |
| Monounsaturated fatty acids | Nightingale Health | 114,999 | European | 2020 | [met-d-MUFA](https://gwas.mrcieu.ac.uk/datasets/met-d-MUFA/) |
| Omega-3 fatty acids | Nightingale Health | 114,999 | European | 2020 | [met-d-Omega_3](https://gwas.mrcieu.ac.uk/datasets/met-d-Omega_3/) |
| Omega-6 fatty acids | Nightingale Health | 114,999 | European | 2020 | [met-d-Omega_6](https://gwas.mrcieu.ac.uk/datasets/met-d-Omega_6/) |
| Polyunsaturated fatty acids | Nightingale Health | 114,999 | European | 2020 | [met-d-PUFA](https://gwas.mrcieu.ac.uk/datasets/met-d-PUFA/) |
| Saturated fatty acids | Nightingale Health | 114,999 | European | 2020 | met-d-SFA |
| **Free cholesterol** |  |  |  |  |  |
| Free cholesterol in HDL | Nightingale Health | 115,078 | European | 2020 | [met-d-HDL_FC](https://gwas.mrcieu.ac.uk/datasets/met-d-HDL_FC/) |
| Free cholesterol in IDL | Nightingale Health | 115,078 | European | 2020 | [met-d-IDL_FC](https://gwas.mrcieu.ac.uk/datasets/met-d-IDL_FC/) |
| Free cholesterol in LDL | Nightingale Health | 115,078 | European | 2020 | [met-d-LDL_FC](https://gwas.mrcieu.ac.uk/datasets/met-d-LDL_FC/) |
| Free cholesterol in VLDL | Nightingale Health | 115,078 | European | 2020 | [met-d-VLDL_FC](https://gwas.mrcieu.ac.uk/datasets/met-d-VLDL_FC/) |
| Free cholesterol in chylomicrons and extremely large VLDL | Nightingale Health | 115,078 | European | 2020 | [met-d-XXL_VLDL_FC](https://gwas.mrcieu.ac.uk/datasets/met-d-XXL_VLDL_FC/) |
| Free cholesterol in large HDL | Nightingale Health | 115,078 | European | 2020 | [met-d-L_HDL_FC](https://gwas.mrcieu.ac.uk/datasets/met-d-L_HDL_FC/) |
| Free cholesterol in large LDL | Nightingale Health | 115,078 | European | 2020 | [met-d-L_LDL_FC](https://gwas.mrcieu.ac.uk/datasets/met-d-L_LDL_FC/) |
| Free cholesterol in large VLDL | Nightingale Health | 115,078 | European | 2020 | [met-d-L_VLDL_FC](https://gwas.mrcieu.ac.uk/datasets/met-d-L_VLDL_FC/) |
| Free cholesterol in medium HDL | Nightingale Health | 115,078 | European | 2020 | [met-d-M_HDL_FC](https://gwas.mrcieu.ac.uk/datasets/met-d-M_HDL_FC/) |
| Free cholesterol in medium LDL | Nightingale Health | 115,078 | European | 2020 | [met-d-M_LDL_FC](https://gwas.mrcieu.ac.uk/datasets/met-d-M_LDL_FC/) |
| Free cholesterol in medium VLDL | Nightingale Health | 115,078 | European | 2020 | [met-d-M_VLDL_FC](https://gwas.mrcieu.ac.uk/datasets/met-d-M_VLDL_FC/) |
| Free cholesterol in small HDL | Nightingale Health | 115,078 | European | 2020 | [met-d-S_HDL_FC](https://gwas.mrcieu.ac.uk/datasets/met-d-S_HDL_FC/) |
| Free cholesterol in small LDL | Nightingale Health | 115,078 | European | 2020 | [met-d-S_LDL_FC](https://gwas.mrcieu.ac.uk/datasets/met-d-S_LDL_FC/) |
| Free cholesterol in small VLDL | Nightingale Health | 115,078 | European | 2020 | [met-d-S_VLDL_FC](https://gwas.mrcieu.ac.uk/datasets/met-d-S_VLDL_FC/) |
| Free cholesterol in very large HDL | Nightingale Health | 115,078 | European | 2020 | [met-d-XL_HDL_FC](https://gwas.mrcieu.ac.uk/datasets/met-d-XL_HDL_FC/) |
| Free cholesterol in very large VLDL | Nightingale Health | 115,078 | European | 2020 | [met-d-XL_VLDL_FC](https://gwas.mrcieu.ac.uk/datasets/met-d-XL_VLDL_FC/) |
| Free cholesterol in very small VLDL | Nightingale Health | 115,078 | European | 2020 | [met-d-XS_VLDL_FC](https://gwas.mrcieu.ac.uk/datasets/met-d-XS_VLDL_FC/) |
| **Glycolysis** |  |  |  |  |  |
| Citrate | Nightingale Health | 115,064 | European | 2020 | [met-d-Citrate](https://gwas.mrcieu.ac.uk/datasets/met-d-Citrate/) |
| Glucose | Nightingale Health | 114,867 | European | 2020 | met-d-Glucose |
| Lactate | Nightingale Health | 114,802 | European | 2020 | met-d-Lactate |
| **Ketone bodies** |  |  |  |  |  |
| 3-Hydroxybutyrate | Nightingale Health | 113,595 | European | 2020 | met-d-bOHbutyrate |
| Acetate | Nightingale Health | 115,046 | European | 2020 | met-d-Acetate |
| Acetoacetate | Nightingale Health | 115,075 | European | 2020 | met-d-Acetoacetate |
| Acetone | Nightingale Health | 115,075 | European | 2020 | met-d-Acetone |
| Albumin | Nightingale Health | 115,060 | European | 2020 | met-d-Albumin |
| Creatinine | Nightingale Health | 110,058 | European | 2020 | met-d-Creatinine |
| Glycoprotein acetyls | Nightingale Health | 115,078 | European | 2020 | met-d-GlycA |
| Pyruvate | Nightingale Health | 114,748 | European | 2020 | met-d-Pyruvate |
| **Lipoprotein particles** |  |  |  |  |  |
| Concentration of HDL particles | Nightingale Health | 115,078 | European | 2020 | met-d-HDL_P |
| Concentration of IDL particles | Nightingale Health | 115,078 | European | 2020 | met-d-IDL_P |
| Concentration of LDL particles | Nightingale Health | 115,078 | European | 2020 | met-d-LDL_P |
| Concentration of VLDL particles | Nightingale Health | 115,078 | European | 2020 | met-d-VLDL_P |
| Concentration of chylomicrons and extremely large VLDL particles | Nightingale Health | 115,078 | European | 2020 | met-d-XXL_VLDL_P |
| Concentration of large HDL particles | Nightingale Health | 115,078 | European | 2020 | met-d-L_HDL_P |
| Concentration of large LDL particles | Nightingale Health | 115,078 | European | 2020 | met-d-L_LDL_P |
| Concentration of large VLDL particles | Nightingale Health | 115,078 | European | 2020 | met-d-L_VLDL_P |
| Concentration of medium HDL particles | Nightingale Health | 115,078 | European | 2020 | met-d-M_HDL_P |
| Concentration of medium LDL particles | Nightingale Health | 115,078 | European | 2020 | met-d-M_LDL_P |
| Concentration of medium VLDL particles | Nightingale Health | 115,078 | European | 2020 | met-d-M_VLDL_P |
| Concentration of small HDL particles | Nightingale Health | 115,078 | European | 2020 | met-d-S_HDL_P |
| Concentration of small LDL particles | Nightingale Health | 115,078 | European | 2020 | met-d-S_LDL_P |
| Concentration of small VLDL particles | Nightingale Health | 115,078 | European | 2020 | met-d-S_VLDL_P |
| Concentration of very large HDL particles | Nightingale Health | 115,078 | European | 2020 | met-d-XL_HDL_P |
| Concentration of very large VLDL particles | Nightingale Health | 115,078 | European | 2020 | met-d-XL_VLDL_P |
| Concentration of very small VLDL particles | Nightingale Health | 115,078 | European | 2020 | met-d-XS_VLDL_P |
| **Phospholipids** |  |  |  |  |  |
| Phospholipids in HDL | Nightingale Health | 115,078 | European | 2020 | met-d-HDL_PL |
| Phospholipids in IDL | Nightingale Health | 115,078 | European | 2020 | met-d-IDL_PL |
| Phospholipids in LDL | Nightingale Health | 115,078 | European | 2020 | met-d-LDL_PL |
| Phospholipids in VLDL | Nightingale Health | 115,078 | European | 2020 | met-d-VLDL_PL |
| Phospholipids in chylomicrons and extremely large VLDL | Nightingale Health | 115,078 | European | 2020 | met-d-XXL_VLDL_PL |
| Phospholipids in large HDL | Nightingale Health | 115,078 | European | 2020 | met-d-L_HDL_PL |
| Phospholipids in large LDL | Nightingale Health | 115,078 | European | 2020 | met-d-L_LDL_PL |
| Phospholipids in large VLDL | Nightingale Health | 115,078 | European | 2020 | met-d-L_VLDL_PL |
| Phospholipids in medium HDL | Nightingale Health | 115,078 | European | 2020 | met-d-M_HDL_PL |
| Phospholipids in medium LDL | Nightingale Health | 115,078 | European | 2020 | met-d-M_LDL_PL |
| Phospholipids in medium VLDL | Nightingale Health | 115,078 | European | 2020 | met-d-M_VLDL_PL |
| Phospholipids in small HDL | Nightingale Health | 115,078 | European | 2020 | met-d-S_HDL_PL |
| Phospholipids in small LDL | Nightingale Health | 115,078 | European | 2020 | met-d-S_LDL_PL |
| Phospholipids in small VLDL | Nightingale Health | 115,078 | European | 2020 | met-d-S_VLDL_PL |
| Phospholipids in very large HDL | Nightingale Health | 115,078 | European | 2020 | met-d-XL_HDL_PL |
| Phospholipids in very large VLDL | Nightingale Health | 115,078 | European | 2020 | met-d-XL_VLDL_PL |
| Phospholipids in very small VLDL | Nightingale Health | 115,078 | European | 2020 | met-d-XS_VLDL_PL |
| **Size&Apo-LP** |  |  |  |  |  |
| Apolipoprotein A1 | Nightingale Health | 115,078 | European | 2020 | met-d-ApoA1 |
| Apolipoprotein B | Nightingale Health | 115,078 | European | 2020 | met-d-ApoB |
| Average diameter for HDL particles | Nightingale Health | 115,078 | European | 2020 | met-d-HDL_size |
| Average diameter for LDL particles | Nightingale Health | 115,078 | European | 2020 | met-d-LDL_size |
| Average diameter for VLDL particles | Nightingale Health | 115,078 | European | 2020 | met-d-VLDL_size |
| **Total lipids** |  |  |  |  |  |
| Total lipids in HDL | Nightingale Health | 115,078 | European | 2020 | met-d-HDL_L |
| Total lipids in IDL | Nightingale Health | 115,078 | European | 2020 | met-d-IDL_L |
| Total lipids in LDL | Nightingale Health | 115,078 | European | 2020 | met-d-LDL_L |
| Total lipids in VLDL | Nightingale Health | 115,078 | European | 2020 | met-d-VLDL_L |
| Total lipids in chylomicrons and extremely large VLDL | Nightingale Health | 115,078 | European | 2020 | met-d-XXL_VLDL_L |
| Total lipids in large HDL | Nightingale Health | 115,078 | European | 2020 | met-d-L_HDL_L |
| Total lipids in large LDL | Nightingale Health | 115,078 | European | 2020 | met-d-L_LDL_L |
| Total lipids in large VLDL | Nightingale Health | 115,078 | European | 2020 | met-d-L_VLDL_L |
| Total lipids in lipoprotein particles | Nightingale Health | 115,078 | European | 2020 | met-d-Total_L |
| Total lipids in medium HDL | Nightingale Health | 115,078 | European | 2020 | met-d-M_HDL_L |
| Total lipids in medium LDL | Nightingale Health | 115,078 | European | 2020 | met-d-M_LDL_L |
| Total lipids in medium VLDL | Nightingale Health | 115,078 | European | 2020 | met-d-M_VLDL_L |
| Total lipids in small HDL | Nightingale Health | 115,078 | European | 2020 | met-d-S_HDL_L |
| Total lipids in small LDL | Nightingale Health | 115,078 | European | 2020 | met-d-S_LDL_L |
| Total lipids in small VLDL | Nightingale Health | 115,078 | European | 2020 | met-d-S_VLDL_L |
| Total lipids in very large HDL | Nightingale Health | 115,078 | European | 2020 | met-d-XL_HDL_L |
| Total lipids in very large VLDL | Nightingale Health | 115,078 | European | 2020 | met-d-XL_VLDL_L |
| Total lipids in very small VLDL | Nightingale Health | 115,078 | European | 2020 | met-d-XS_VLDL_L |
| **Triglycerides** |  |  |  |  |  |
| Triglycerides in HDL | Nightingale Health | 115,078 | European | 2020 | met-d-HDL_TG |
| Triglycerides in IDL | Nightingale Health | 115,078 | European | 2020 | met-d-IDL_TG |
| Triglycerides in LDL | Nightingale Health | 115,078 | European | 2020 | met-d-LDL_TG |
| Triglycerides in VLDL | Nightingale Health | 115,078 | European | 2020 | met-d-VLDL_TG |
| Triglycerides in chylomicrons and extremely large VLDL | Nightingale Health | 115,078 | European | 2020 | met-d-XXL_VLDL_TG |
| Triglycerides in large HDL | Nightingale Health | 115,078 | European | 2020 | met-d-L_HDL_TG |
| Triglycerides in large LDL | Nightingale Health | 115,078 | European | 2020 | met-d-L_LDL_TG |
| Triglycerides in large VLDL | Nightingale Health | 115,078 | European | 2020 | met-d-L_VLDL_TG |
| Triglycerides in medium HDL | Nightingale Health | 115,078 | European | 2020 | met-d-M_HDL_TG |
| Triglycerides in medium LDL | Nightingale Health | 115,078 | European | 2020 | met-d-M_LDL_TG |
| Triglycerides in medium VLDL | Nightingale Health | 115,078 | European | 2020 | met-d-M_VLDL_TG |
| Triglycerides in small HDL | Nightingale Health | 115,078 | European | 2020 | met-d-S_HDL_TG |
| Triglycerides in small LDL | Nightingale Health | 115,078 | European | 2020 | met-d-S_LDL_TG |
| Triglycerides in small VLDL | Nightingale Health | 115,078 | European | 2020 | met-d-S_VLDL_TG |
| Triglycerides in very large HDL | Nightingale Health | 115,078 | European | 2020 | met-d-XL_HDL_TG |
| Triglycerides in very large VLDL | Nightingale Health | 115,078 | European | 2020 | met-d-XL_VLDL_TG |
| Triglycerides in very small VLDL | Nightingale Health | 115,078 | European | 2020 | met-d-XS_VLDL_TG |

| **Supplementary Table 2. Instrumental variables for SGLT2 inhibition** | | | | | | | | | | | |
| --- | --- | --- | --- | --- | --- | --- | --- | --- | --- | --- | --- |
| SNP | Effect_allele | Other_allele | Beta | Se | *P* | Samplesize | Effect allele frequency | R2-variance explained | Sum_R2 | F-statistics | Overall_F-statistics |
| rs111510548 | C | T | 0.015 | 0.004 | 6.69E-05 | 344182 | 0.103 | 4.29E-05 |  | 15.897 |  |
| rs8057207 | T | C | 0.013 | 0.002 | 4.55E-08 | 344182 | 0.362 | 7.91E-05 |  | 29.900 |  |
| rs9926717 | G | A | 0.011 | 0.003 | 9.61E-06 | 344182 | 0.284 | 5.22E-05 |  | 19.588 |  |
| rs116943658 | A | G | 0.013 | 0.002 | 3.01E-07 | 344182 | 0.330 | 6.99E-05 |  | 26.244 |  |
| rs13334492 | A | G | 0.011 | 0.002 | 7.57E-07 | 344182 | 0.442 | 6.48E-05 |  | 24.463 |  |
| rs2070896 | C | T | 0.017 | 0.002 | 1.70E-12 | 344182 | 0.375 | 1.40E-04 |  | 49.808 |  |
| rs28641848 | T | C | 0.011 | 0.003 | 1.21E-05 | 344182 | 0.279 | 5.09E-05 |  | 19.151 |  |
| rs28692853 | A | C | 0.015 | 0.002 | 2.78E-10 | 344182 | 0.507 | 1.06E-04 |  | 39.827 |  |
| rs67464975 | T | C | 0.012 | 0.002 | 4.03E-07 | 344182 | 0.470 | 6.84E-05 |  | 25.682 |  |
| rs8050328 | G | T | 0.016 | 0.002 | 1.09E-11 | 344182 | 0.364 | 1.23E-04 | 7.96E-04 | 46.152 | 27.434 |

**Supplementary Table 3. MR estimates of the effect of SGLT2 inhibition on circulating metabolites**

| Circulating metabolites | Method | β (95% CI) | *P* | Q statistic | *P* -heterogeneity | Egger intercept | *P* -intercept |
| --- | --- | --- | --- | --- | --- | --- | --- |
| **Amino acids** |  |  |  |  |  |  |  |
| Alanine | IVW | 0.34 (0.14, 0.54) | 0.001 | 6.040 | 0.736 |  |  |
|  | MR-Egger | 0.05 (-1.26, 1.36) | 0.943 | 5.848 | 0.664 | -0.004 | 0.672 |
|  | MR-PRESSO | 0.34 (0.17, 0.50) | 0.003 | 7.090 | 0.790 |  |  |
| Glutamine | IVW | -0.03 (-0.24, 0.17) | 0.765 | 3.944 | 0.915 |  |  |
|  | MR-Egger | -0.95 (-2.27, 0.37) | 0.194 | 2.020 | 0.980 | -0.013 | 0.203 |
|  | MR-PRESSO | -0.03 (-0.17, 0.10) | 0.662 | 5.131 | 0.903 |  |  |
| Glycine | IVW | 0.34 (0.15, 0.54) | 4.91e-04 | 6.655 | 0.673 |  |  |
|  | MR-Egger | -0.87 (-2.12, 0.37) | 0.207 | 2.902 | 0.940 | -0.017 | 0.089 |
|  | MR-PRESSO | 0.34 (0.18, 0.51) | 0.003 | 8.185 | 0.704 |  |  |
| Histidine | IVW | -0.32 (-0.53, -0.11) | 0.002 | 2.479 | 0.981 |  |  |
|  | MR-Egger | -0.45 (-1.77, 0.88) | 0.526 | 2.442 | 0.964 | -0.002 | 0.852 |
|  | MR-PRESSO | -0.32 (-0.43, -0.21) | 2.56e-04 | 3.062 | 0.980 |  |  |
| Isoleucine | IVW | -0.34 (-0.54, -0.14) | 0.001 | 2.282 | 0.986 |  |  |
|  | MR-Egger | 0.12 (-1.17, 1.42) | 0.856 | 1.772 | 0.987 | 0.006 | 0.495 |
|  | MR-PRESSO | -0.34 (-0.44, -0.24) | 9.52e-05 | 2.721 | 0.987 |  |  |
| Leucine | IVW | -0.24 (-0.44, -0.05) | 0.015 | 2.053 | 0.991 |  |  |
|  | MR-Egger | -0.17 (-1.43, 1.10) | 0.799 | 2.039 | 0.980 | 0.001 | 0.909 |
|  | MR-PRESSO | -0.24 (-0.34, -0.15) | 0.001 | 2.481 | 0.992 |  |  |
| Phenylalanine | IVW | -0.26 (-0.46, -0.05) | 0.014 | 3.508 | 0.941 |  |  |
|  | MR-Egger | -0.62 (-1.94, 0.70) | 0.387 | 3.215 | 0.920 | -0.005 | 0.603 |
|  | MR-PRESSO | -0.26 (-0.38, -0.13) | 0.003 | 4.161 | 0.953 |  |  |
| Tyrosine | IVW | -0.03 (-0.23, 0.18) | 0.799 | 4.889 | 0.844 |  |  |
|  | MR-Egger | -0.16 (-1.48, 1.16) | 0.820 | 4.850 | 0.774 | -0.002 | 0.848 |
|  | MR-PRESSO | -0.03 (-0.18, 0.12) | 0.738 | 5.839 | 0.861 |  |  |
| Valine | IVW | 0.07 (-0.13, 0.27) | 0.512 | 3.197 | 0.956 |  |  |
|  | MR-Egger | 0.09 (-1.19, 1.37) | 0.895 | 3.196 | 0.921 | 0.000 | 0.973 |
|  | MR-PRESSO | 0.07 (-0.05, 0.19) | 0.300 | 3.878 | 0.958 |  |  |
| **Cholesterol** |  |  |  |  |  |  |  |
| Cholesterol in IDL | IVW | 0.06 (-0.14, 0.26) | 0.554 | 6.423 | 0.697 |  |  |
|  | MR-Egger | -1.06 (-2.33, 0.22) | 0.142 | 3.393 | 0.907 | -0.015 | 0.120 |
|  | MR-PRESSO | 0.06 (-0.11, 0.23) | 0.502 | 8.411 | 0.661 |  |  |
| Cholesterol in chylomicrons and extremely large VLDL | IVW | 0.19 (0, 0.39) | 0.055 | 1.714 | 0.995 |  |  |
|  | MR-Egger | 0.53 (-0.74, 1.81) | 0.436 | 1.434 | 0.994 | 0.005 | 0.611 |
|  | MR-PRESSO | 0.19 (0.11, 0.28) | 0.002 | 2.181 | 0.994 |  |  |
| Cholesterol in large HDL | IVW | -0.22 (-0.41, -0.02) | 0.033 | 11.010 | 0.275 |  |  |
|  | MR-Egger | -0.69 (-2.01, 0.62) | 0.331 | 10.335 | 0.242 | -0.007 | 0.490 |
|  | MR-PRESSO | -0.22 (-0.41, -0.02) | 0.062 | 13.659 | 0.295 |  |  |
| Cholesterol in large LDL | IVW | 0.24 (0.04, 0.44) | 0.019 | 6.563 | 0.682 |  |  |
|  | MR-Egger | -1.03 (-2.34, 0.27) | 0.160 | 2.821 | 0.945 | -0.017 | 0.089 |
|  | MR-PRESSO | 0.24 (0.07, 0.42) | 0.023 | 8.599 | 0.670 |  |  |
| Cholesterol in large VLDL | IVW | 0.18 (-0.02, 0.38) | 0.072 | 0.924 | 1.000 |  |  |
|  | MR-Egger | 0.25 (-1.04, 1.54) | 0.711 | 0.913 | 0.999 | 0.001 | 0.918 |
|  | MR-PRESSO | 0.18 (0.12, 0.25) | 3.25e-04 | 1.132 | 1.000 |  |  |
| Cholesterol in medium HDL | IVW | 0.30 (0.11, 0.48) | 0.002 | 5.883 | 0.752 |  |  |
|  | MR-Egger | -0.70 (-1.91, 0.52) | 0.293 | 3.251 | 0.918 | -0.014 | 0.143 |
|  | MR-PRESSO | 0.30 (0.14, 0.45) | 0.004 | 7.898 | 0.729 |  |  |
| Cholesterol in medium LDL | IVW | 0.23 (0.03, 0.44) | 0.026 | 3.185 | 0.956 |  |  |
|  | MR-Egger | -0.74 (-2.06, 0.58) | 0.305 | 1.053 | 0.998 | -0.013 | 0.182 |
|  | MR-PRESSO | 0.23 (0.11, 0.36) | 0.005 | 4.124 | 0.957 |  |  |
| Cholesterol in medium VLDL | IVW | 0.04 (-0.17, 0.24) | 0.732 | 3.008 | 0.964 |  |  |
|  | MR-Egger | -0.80 (-2.12, 0.51) | 0.267 | 1.415 | 0.994 | -0.011 | 0.242 |
|  | MR-PRESSO | 0.04 (-0.08, 0.15) | 0.569 | 3.814 | 0.953 |  |  |
| Cholesterol in small HDL | IVW | 0.65 (0.44, 0.85) | 6.06e-10 | 6.708 | 0.668 |  |  |
|  | MR-Egger | -0.78 (-2.09, 0.54) | 0.281 | 2.120 | 0.977 | -0.020 | 0.065 |
|  | MR-PRESSO | 0.65 (0.47, 0.82) | 5.26e-05 | 8.733 | 0.715 |  |  |
| Cholesterol in small LDL | IVW | 0.16 (-0.05, 0.36) | 0.132 | 2.957 | 0.966 |  |  |
|  | MR-Egger | -0.75 (-2.07, 0.58) | 0.301 | 1.117 | 0.997 | -0.012 | 0.212 |
|  | MR-PRESSO | 0.16 (0.04, 0.27) | 0.028 | 3.810 | 0.956 |  |  |
| Cholesterol in small VLDL | IVW | 0.16 (-0.04, 0.37) | 0.121 | 1.376 | 0.998 |  |  |
|  | MR-Egger | -0.45 (-1.77, 0.87) | 0.525 | 0.536 | 1.000 | -0.008 | 0.386 |
|  | MR-PRESSO | 0.16 (0.08, 0.24) | 0.003 | 1.671 | 0.997 |  |  |
| Cholesterol in very large HDL | IVW | -0.52 (-0.76, -0.29) | 1.21e-05 | 14.490 | 0.106 |  |  |
|  | MR-Egger | -0.68 (-2.27, 0.92) | 0.429 | 14.423 | 0.071 | -0.002 | 0.852 |
|  | MR-PRESSO | -0.52 (-0.76, -0.29) | 0.002 | 17.548 | 0.172 |  |  |
| Cholesterol in very large VLDL | IVW | 0.19 (-0.01, 0.39) | 0.062 | 0.817 | 1.000 |  |  |
|  | MR-Egger | 0.24 (-1.05, 1.53) | 0.721 | 0.810 | 0.999 | 0.001 | 0.937 |
|  | MR-PRESSO | 0.19 (0.13, 0.25) | 1.60e-04 | 1.011 | 1.000 |  |  |
| Cholesterol in very small VLDL | IVW | -0.04 (-0.23, 0.16) | 0.725 | 3.170 | 0.957 |  |  |
|  | MR-Egger | -0.72 (-2.00, 0.56) | 0.305 | 2.057 | 0.979 | -0.009 | 0.322 |
|  | MR-PRESSO | -0.04 (-0.15, 0.08) | 0.567 | 4.060 | 0.948 |  |  |
| **Cholines** |  |  |  |  |  |  |  |
| Phosphatidylcholines | IVW | 0.37 (0.17, 0.56) | 1.91e-04 | 3.757 | 0.927 |  |  |
|  | MR-Egger | -0.48 (-1.71, 0.76) | 0.473 | 1.937 | 0.983 | -0.012 | 0.214 |
|  | MR-PRESSO | 0.37 (0.24, 0.49) | 2.68e-04 | 5.046 | 0.930 |  |  |
| Phosphoglycerides | IVW | 0.34 (0.15, 0.54) | 0.001 | 4.048 | 0.908 |  |  |
|  | MR-Egger | -0.51 (-1.76, 0.74) | 0.443 | 2.202 | 0.974 | -0.012 | 0.211 |
|  | MR-PRESSO | 0.34 (0.21, 0.47) | 0.001 | 5.441 | 0.911 |  |  |
| Sphingomyelins | IVW | 0.12 (-0.08, 0.33) | 0.241 | 10.375 | 0.321 |  |  |
|  | MR-Egger | -1.2 (-2.43, 0.03) | 0.093 | 5.850 | 0.664 | -0.018 | 0.066 |
|  | MR-PRESSO | 0.12 (-0.08, 0.33) | 0.271 | 13.657 | 0.274 |  |  |
| Total cholines | IVW | 0.32 (0.13, 0.51) | 0.001 | 5.275 | 0.810 |  |  |
|  | MR-Egger | -0.67 (-1.91, 0.57) | 0.318 | 2.750 | 0.949 | -0.014 | 0.151 |
|  | MR-PRESSO | 0.32 (0.17, 0.47) | 0.002 | 7.064 | 0.783 |  |  |
| **Compounds** |  |  |  |  |  |  |  |
| Clinical LDL cholesterol | IVW | 0.15 (-0.05, 0.35) | 0.152 | 5.365 | 0.801 |  |  |
|  | MR-Egger | -1.02 (-2.33, 0.29) | 0.167 | 2.253 | 0.972 | -0.016 | 0.116 |
|  | MR-PRESSO | 0.15 (-0.01, 0.31) | 0.097 | 6.998 | 0.779 |  |  |
| HDL cholesterol | IVW | 0.09 (-0.09, 0.28) | 0.327 | 9.339 | 0.407 |  |  |
|  | MR-Egger | -0.81 (-1.98, 0.37) | 0.214 | 7.019 | 0.535 | -0.012 | 0.166 |
|  | MR-PRESSO | 0.09 (-0.09, 0.28) | 0.353 | 12.111 | 0.414 |  |  |
| LDL cholesterol | IVW | 0.23 (0.03, 0.44) | 0.025 | 5.177 | 0.819 |  |  |
|  | MR-Egger | -0.94 (-2.25, 0.37) | 0.198 | 2.035 | 0.980 | -0.016 | 0.114 |
|  | MR-PRESSO | 0.23 (0.08, 0.39) | 0.016 | 6.774 | 0.813 |  |  |
| Remnant cholesterol (non-HDL, non-LDL -cholesterol) | IVW | 0.09 (-0.11, 0.30) | 0.376 | 2.853 | 0.970 |  |  |
|  | MR-Egger | -0.74 (-2.05, 0.57) | 0.302 | 1.276 | 0.996 | -0.011 | 0.245 |
|  | MR-PRESSO | 0.09 (-0.02, 0.21) | 0.150 | 3.701 | 0.967 |  |  |
| Total cholesterol | IVW | 0.18 (-0.02, 0.37) | 0.083 | 6.661 | 0.672 |  |  |
|  | MR-Egger | -0.98 (-2.26, 0.29) | 0.169 | 3.412 | 0.906 | -0.016 | 0.109 |
|  | MR-PRESSO | 0.18 (0.01, 0.35) | 0.074 | 8.751 | 0.625 |  |  |
| Total cholesterol minus HDL-C | IVW | 0.17 (-0.03, 0.37) | 0.101 | 4.001 | 0.911 |  |  |
|  | MR-Egger | -0.85 (-2.16, 0.47) | 0.241 | 1.634 | 0.990 | -0.014 | 0.163 |
|  | MR-PRESSO | 0.17 (0.03, 0.31) | 0.036 | 5.222 | 0.901 |  |  |
| Total concentration of branched-chain amino acids (leucine + isoleucine + valine | IVW | -0.12 (-0.31, 0.08) | 0.247 | 2.321 | 0.985 |  |  |
|  | MR-Egger | 0 (-1.28, 1.27) | 0.998 | 2.289 | 0.971 | 0.002 | 0.862 |
|  | MR-PRESSO | -0.12 (-0.22, -0.02) | 0.049 | 2.811 | 0.992 |  |  |
| Total concentration of lipoprotein particles | IVW | 0.42 (0.22, 0.61) | 2.23e-05 | 6.913 | 0.646 |  |  |
|  | MR-Egger | -0.87 (-2.11, 0.37) | 0.207 | 2.691 | 0.952 | -0.018 | 0.074 |
|  | MR-PRESSO | 0.42 (0.25, 0.59) | 0.001 | 9.364 | 0.625 |  |  |
| Total esterified cholesterol | IVW | 0.19 (-0.01, 0.39) | 0.061 | 7.261 | 0.610 |  |  |
|  | MR-Egger | -1.02 (-2.30, 0.25) | 0.154 | 3.695 | 0.884 | -0.017 | 0.096 |
|  | MR-PRESSO | 0.19 (0.01, 0.37) | 0.067 | 9.554 | 0.578 |  |  |
| Total free cholesterol | IVW | 0.14 (-0.06, 0.34) | 0.166 | 5.135 | 0.822 |  |  |
|  | MR-Egger | -0.88 (-2.17, 0.41) | 0.219 | 2.681 | 0.953 | -0.014 | 0.156 |
|  | MR-PRESSO | 0.14 (-0.01, 0.29) | 0.100 | 6.714 | 0.815 |  |  |
| Total phospholipids in lipoprotein particles | IVW | 0.25 (0.06, 0.45) | 0.012 | 4.641 | 0.864 |  |  |
|  | MR-Egger | -0.65 (-1.91, 0.60) | 0.339 | 2.610 | 0.956 | -0.012 | 0.192 |
|  | MR-PRESSO | 0.25 (0.11, 0.39) | 0.007 | 6.171 | 0.846 |  |  |
| Total triglycerides | IVW | 0.29 (0.09, 0.49) | 0.005 | 1.193 | 0.999 |  |  |
|  | MR-Egger | 0.48 (-0.79, 1.76) | 0.479 | 1.099 | 0.998 | 0.003 | 0.768 |
|  | MR-PRESSO | 0.29 (0.22, 0.36) | 2.70e-05 | 1.487 | 0.999 |  |  |
| VLDL cholesterol | IVW | 0.12 (-0.09, 0.32) | 0.259 | 0.681 | 1.000 |  |  |
|  | MR-Egger | -0.30 (-1.62, 1.02) | 0.669 | 0.289 | 1.000 | -0.006 | 0.549 |
|  | MR-PRESSO | 0.12 (0.06, 0.17) | 0.003 | 0.830 | 1.000 |  |  |
| **Esterified cholesterol** |  |  |  |  |  |  |  |
| Cholesteryl esters in HDL | IVW | 0.11 (-0.08, 0.29) | 0.269 | 9.347 | 0.406 |  |  |
|  | MR-Egger | -0.86 (-2.04, 0.32) | 0.193 | 6.733 | 0.566 | -0.013 | 0.145 |
|  | MR-PRESSO | 0.11 (-0.08, 0.29) | 0.298 | 12.179 | 0.366 |  |  |
| Cholesteryl esters in IDL | IVW | 0.08 (-0.12, 0.28) | 0.418 | 5.966 | 0.743 |  |  |
|  | MR-Egger | -1.02 (-2.29, 0.25) | 0.155 | 3.021 | 0.933 | -0.015 | 0.125 |
|  | MR-PRESSO | 0.08 (-0.08, 0.24) | 0.345 | 7.812 | 0.712 |  |  |
| Cholesteryl esters in LDL | IVW | 0.26 (0.06, 0.47) | 0.011 | 4.549 | 0.872 |  |  |
|  | MR-Egger | -0.87 (-2.18, 0.45) | 0.233 | 1.642 | 0.990 | -0.016 | 0.127 |
|  | MR-PRESSO | 0.26 (0.12, 0.41) | 0.006 | 5.959 | 0.868 |  |  |
| Cholesteryl esters in VLDL | IVW | 0.09 (-0.11, 0.30) | 0.375 | 1.170 | 0.999 |  |  |
|  | MR-Egger | -0.46 (-1.78, 0.86) | 0.512 | 0.475 | 1.000 | -0.008 | 0.429 |
|  | MR-PRESSO | 0.09 (0.02, 0.17) | 0.036 | 1.445 | 0.998 |  |  |
| Cholesteryl esters in chylomicrons and extremely large VLDL | IVW | 0.20 (0.01, 0.40) | 0.043 | 1.285 | 0.998 |  |  |
|  | MR-Egger | 0.42 (-0.85, 1.70) | 0.534 | 1.169 | 0.997 | 0.003 | 0.742 |
|  | MR-PRESSO | 0.20 (0.13, 0.28) | 4.66e-04 | 1.615 | 0.998 |  |  |
| Cholesteryl esters in large HDL | IVW | -0.20 (-0.41, 0) | 0.045 | 11.114 | 0.268 |  |  |
|  | MR-Egger | -0.73 (-2.05, 0.59) | 0.310 | 10.312 | 0.244 | -0.007 | 0.453 |
|  | MR-PRESSO | -0.20 (-0.41, 0) | 0.076 | 13.829 | 0.266 |  |  |
| Cholesteryl esters in large LDL | IVW | 0.28 (0.08, 0.48) | 0.007 | 6.120 | 0.728 |  |  |
|  | MR-Egger | -0.98 (-2.29, 0.32) | 0.179 | 2.439 | 0.964 | -0.017 | 0.091 |
|  | MR-PRESSO | 0.28 (0.11, 0.45) | 0.009 | 8.031 | 0.730 |  |  |
| Cholesteryl esters in large VLDL | IVW | 0.14 (-0.07, 0.34) | 0.190 | 0.658 | 1.000 |  |  |
|  | MR-Egger | 0.10 (-1.21, 1.40) | 0.890 | 0.655 | 1.000 | -0.001 | 0.953 |
|  | MR-PRESSO | 0.14 (0.08, 0.19) | 0.001 | 0.782 | 1.000 |  |  |
| Cholesteryl esters in medium HDL | IVW | 0.30 (0.12, 0.49) | 0.002 | 5.842 | 0.756 |  |  |
|  | MR-Egger | -0.72 (-1.94, 0.49) | 0.278 | 3.040 | 0.932 | -0.014 | 0.133 |
|  | MR-PRESSO | 0.30 (0.15, 0.46) | 0.004 | 7.867 | 0.733 |  |  |
| Cholesteryl esters in medium LDL | IVW | 0.24 (0.03, 0.44) | 0.023 | 2.439 | 0.982 |  |  |
|  | MR-Egger | -0.62 (-1.94, 0.71) | 0.388 | 0.794 | 0.999 | -0.012 | 0.236 |
|  | MR-PRESSO | 0.24 (0.13, 0.34) | 0.002 | 3.138 | 0.982 |  |  |
| Cholesteryl esters in medium VLDL | IVW | -0.02 (-0.22, 0.18) | 0.835 | 4.523 | 0.874 |  |  |
|  | MR-Egger | -0.97 (-2.28, 0.34) | 0.184 | 2.450 | 0.964 | -0.013 | 0.188 |
|  | MR-PRESSO | -0.02 (-0.17, 0.12) | 0.776 | 5.724 | 0.870 |  |  |
| Cholesteryl esters in small HDL | IVW | 0.64 (0.43, 0.84) | 1.17e-09 | 6.967 | 0.641 |  |  |
|  | MR-Egger | -0.79 (-2.11, 0.53) | 0.274 | 2.362 | 0.968 | -0.020 | 0.064 |
|  | MR-PRESSO | 0.64 (0.46, 0.82) | 6.95e-05 | 8.981 | 0.697 |  |  |
| Cholesteryl esters in small LDL | IVW | 0.18 (-0.02, 0.39) | 0.080 | 2.386 | 0.984 |  |  |
|  | MR-Egger | -0.65 (-1.97, 0.67) | 0.361 | 0.808 | 0.999 | -0.011 | 0.244 |
|  | MR-PRESSO | 0.18 (0.08, 0.29) | 0.008 | 3.068 | 0.977 |  |  |
| Cholesteryl esters in small VLDL | IVW | 0.16 (-0.04, 0.37) | 0.121 | 1.226 | 0.999 |  |  |
|  | MR-Egger | -0.35 (-1.66, 0.97) | 0.621 | 0.642 | 1.000 | -0.007 | 0.467 |
|  | MR-PRESSO | 0.16 (0.09, 0.24) | 0.002 | 1.460 | 0.999 |  |  |
| Cholesteryl esters in very large HDL | IVW | -0.47 (-0.70, -0.25) | 4.53e-05 | 13.829 | 0.129 |  |  |
|  | MR-Egger | -0.69 (-2.23, 0.85) | 0.404 | 13.690 | 0.090 | -0.003 | 0.783 |
|  | MR-PRESSO | -0.47 (-0.70, -0.25) | 0.003 | 16.801 | 0.196 |  |  |
| Cholesteryl esters in very large VLDL | IVW | 0.17 (-0.03, 0.38) | 0.090 | 0.779 | 1.000 |  |  |
|  | MR-Egger | 0.09 (-1.21, 1.39) | 0.899 | 0.761 | 0.999 | -0.001 | 0.897 |
|  | MR-PRESSO | 0.17 (0.11, 0.23) | 2.75e-04 | 0.934 | 0.999 |  |  |
| Cholesteryl esters in very small VLDL | IVW | -0.03 (-0.23, 0.16) | 0.736 | 3.927 | 0.916 |  |  |
|  | MR-Egger | -0.79 (-2.06, 0.49) | 0.260 | 2.551 | 0.959 | -0.010 | 0.275 |
|  | MR-PRESSO | -0.03 (-0.16, 0.10) | 0.622 | 5.038 | 0.908 |  |  |
| **Fatty Acids** |  |  |  |  |  |  |  |
| Total fatty acids | IVW | 0.33 (0.13, 0.53) | 0.001 | 1.359 | 0.998 |  |  |
|  | MR-Egger | -0.21 (-1.52, 1.1) | 0.765 | 0.694 | 1.000 | -0.007 | 0.439 |
|  | MR-PRESSO | 0.33 (0.25, 0.41) | 1.76e-05 | 1.821 | 0.998 |  |  |
| Degree of unsaturation | IVW | -0.27 (-0.47, -0.08) | 0.006 | 2.131 | 0.989 |  |  |
|  | MR-Egger | -0.62 (-1.87, 0.64) | 0.365 | 1.836 | 0.986 | -0.005 | 0.602 |
|  | MR-PRESSO | -0.27 (-0.37, -0.18) | 3.26e-04 | 2.741 | 0.991 |  |  |
| Docosahexaenoic acid | IVW | -0.01 (-0.21, 0.18) | 0.886 | 2.828 | 0.971 |  |  |
|  | MR-Egger | -0.23 (-1.50, 1.05) | 0.734 | 2.717 | 0.951 | -0.003 | 0.748 |
|  | MR-PRESSO | -0.01 (-0.13, 0.10) | 0.804 | 3.424 | 0.973 |  |  |
| Linoleic acid | IVW | 0.33 (0.13, 0.53) | 0.001 | 3.875 | 0.919 |  |  |
|  | MR-Egger | -0.74 (-2.03, 0.56) | 0.296 | 1.209 | 0.997 | -0.015 | 0.141 |
|  | MR-PRESSO | 0.33 (0.20, 0.46) | 0.001 | 4.980 | 0.918 |  |  |
| Monounsaturated fatty acids | IVW | 0.40 (0.19, 0.60) | 1.29e-04 | 0.664 | 1.000 |  |  |
|  | MR-Egger | 0.06 (-1.25, 1.37) | 0.929 | 0.406 | 1.000 | -0.005 | 0.625 |
|  | MR-PRESSO | 0.40 (0.34, 0.45) | 1.94e-07 | 0.852 | 1.000 |  |  |
| Omega-3 fatty acids | IVW | 0.01 (-0.19, 0.21) | 0.914 | 2.549 | 0.980 |  |  |
|  | MR-Egger | 0.02 (-1.28, 1.32) | 0.973 | 2.549 | 0.959 | 0.000 | 0.986 |
|  | MR-PRESSO | 0.01 (-0.10, 0.12) | 0.844 | 2.956 | 0.985 |  |  |
| Omega-6 fatty acids | IVW | 0.32 (0.12, 0.52) | 0.002 | 3.705 | 0.930 |  |  |
|  | MR-Egger | -0.74 (-2.03, 0.55) | 0.295 | 1.048 | 0.998 | -0.015 | 0.142 |
|  | MR-PRESSO | 0.32 (0.19, 0.45) | 0.001 | 4.948 | 0.912 |  |  |
| Polyunsaturated fatty acids | IVW | 0.27 (0.07, 0.47) | 0.007 | 2.982 | 0.965 |  |  |
|  | MR-Egger | -0.60 (-1.88, 0.69) | 0.390 | 1.184 | 0.997 | -0.012 | 0.217 |
|  | MR-PRESSO | 0.27 (0.16, 0.39) | 0.001 | 3.996 | 0.958 |  |  |
| Saturated fatty acids | IVW | 0.28 (0.07, 0.48) | 0.008 | 1.444 | 0.998 |  |  |
|  | MR-Egger | -0.10 (-1.41, 1.21) | 0.886 | 1.121 | 0.997 | -0.005 | 0.585 |
|  | MR-PRESSO | 0.28 (0.19, 0.36) | 9.28e-05 | 1.889 | 0.996 |  |  |
| **Free cholesterol** |  |  |  |  |  |  |  |
| Free cholesterol in HDL | IVW | 0.05 (-0.14, 0.23) | 0.625 | 8.950 | 0.442 |  |  |
|  | MR-Egger | -0.65 (-1.82, 0.51) | 0.305 | 7.544 | 0.479 | -0.010 | 0.270 |
|  | MR-PRESSO | 0.05 (-0.14, 0.23) | 0.636 | 11.411 | 0.437 |  |  |
| Free cholesterol in IDL | IVW | -0.01 (-0.21, 0.19) | 0.935 | 7.592 | 0.576 |  |  |
|  | MR-Egger | -1.14 (-2.42, 0.13) | 0.118 | 4.486 | 0.811 | -0.016 | 0.116 |
|  | MR-PRESSO | -0.01 (-0.19, 0.17) | 0.931 | 9.923 | 0.542 |  |  |
| Free cholesterol in LDL | IVW | 0.15 (-0.05, 0.35) | 0.146 | 6.862 | 0.652 |  |  |
|  | MR-Egger | -1.10 (-2.41, 0.20) | 0.136 | 3.225 | 0.919 | -0.017 | 0.093 |
|  | MR-PRESSO | 0.15 (-0.03, 0.33) | 0.130 | 8.946 | 0.624 |  |  |
| Free cholesterol in VLDL | IVW | 0.15 (-0.05, 0.36) | 0.142 | 0.341 | 1.000 |  |  |
|  | MR-Egger | -0.08 (-1.39, 1.23) | 0.903 | 0.213 | 1.000 | -0.003 | 0.729 |
|  | MR-PRESSO | 0.15 (0.11, 0.19) | 3.51e-05 | 0.407 | 1.000 |  |  |
| Free cholesterol in chylomicrons and extremely large VLDL | IVW | 0.14 (-0.05, 0.34) | 0.155 | 1.817 | 0.994 |  |  |
|  | MR-Egger | 0.58 (-0.70, 1.85) | 0.401 | 1.361 | 0.995 | 0.006 | 0.519 |
|  | MR-PRESSO | 0.14 (0.05, 0.23) | 0.011 | 2.319 | 0.995 |  |  |
| Free cholesterol in large HDL | IVW | -0.25 (-0.44, -0.05) | 0.013 | 10.737 | 0.294 |  |  |
|  | MR-Egger | -0.58 (-1.90, 0.73) | 0.409 | 10.399 | 0.238 | -0.005 | 0.624 |
|  | MR-PRESSO | -0.25 (-0.44, -0.05) | 0.035 | 13.186 | 0.325 |  |  |
| Free cholesterol in large LDL | IVW | 0.13 (-0.07, 0.33) | 0.195 | 7.737 | 0.561 |  |  |
|  | MR-Egger | -1.14 (-2.43, 0.16) | 0.124 | 3.955 | 0.861 | -0.017 | 0.088 |
|  | MR-PRESSO | 0.13 (-0.05, 0.32) | 0.196 | 10.083 | 0.536 |  |  |
| Free cholesterol in large VLDL | IVW | 0.24 (0.04, 0.43) | 0.020 | 1.391 | 0.998 |  |  |
|  | MR-Egger | 0.38 (-0.90, 1.66) | 0.574 | 1.339 | 0.995 | 0.002 | 0.826 |
|  | MR-PRESSO | 0.24 (0.16, 0.31) | 2.23e-04 | 1.720 | 0.999 |  |  |
| Free cholesterol in medium HDL | IVW | 0.25 (0.06, 0.44) | 0.008 | 5.873 | 0.753 |  |  |
|  | MR-Egger | -0.58 (-1.78, 0.62) | 0.370 | 3.977 | 0.859 | -0.011 | 0.206 |
|  | MR-PRESSO | 0.25 (0.10, 0.40) | 0.010 | 7.765 | 0.722 |  |  |
| Free cholesterol in medium LDL | IVW | 0.21 (0, 0.41) | 0.046 | 5.667 | 0.773 |  |  |
|  | MR-Egger | -1.04 (-2.35, 0.28) | 0.162 | 2.156 | 0.976 | -0.017 | 0.098 |
|  | MR-PRESSO | 0.21 (0.05, 0.37) | 0.033 | 7.382 | 0.753 |  |  |
| Free cholesterol in medium VLDL | IVW | 0.10 (-0.10, 0.31) | 0.320 | 1.398 | 0.998 |  |  |
|  | MR-Egger | -0.52 (-1.83, 0.80) | 0.466 | 0.531 | 1.000 | -0.008 | 0.379 |
|  | MR-PRESSO | 0.10 (0.02, 0.18) | 0.033 | 1.762 | 0.998 |  |  |
| Free cholesterol in small HDL | IVW | 0.57 (0.37, 0.77) | 2.34e-08 | 4.452 | 0.879 |  |  |
|  | MR-Egger | -0.57 (-1.87, 0.72) | 0.412 | 1.379 | 0.995 | -0.016 | 0.118 |
|  | MR-PRESSO | 0.57 (0.43, 0.71) | 2.35e-05 | 5.962 | 0.888 |  |  |
| Free cholesterol in small LDL | IVW | 0.10 (-0.10, 0.31) | 0.318 | 5.060 | 0.829 |  |  |
|  | MR-Egger | -0.95 (-2.27, 0.37) | 0.194 | 2.528 | 0.960 | -0.014 | 0.150 |
|  | MR-PRESSO | 0.10 (-0.05, 0.26) | 0.215 | 6.517 | 0.817 |  |  |
| Free cholesterol in small VLDL | IVW | 0.15 (-0.05, 0.36) | 0.146 | 1.873 | 0.993 |  |  |
|  | MR-Egger | -0.6 (-1.92, 0.72) | 0.399 | 0.596 | 1.000 | -0.010 | 0.291 |
|  | MR-PRESSO | 0.15 (0.06, 0.25) | 0.011 | 2.348 | 0.993 |  |  |
| Free cholesterol in very large HDL | IVW | -0.65 (-0.90, -0.40) | 3.97e-07 | 15.489 | 0.078 |  |  |
|  | MR-Egger | -0.56 (-2.27, 1.14) | 0.535 | 15.471 | 0.051 | 0.001 | 0.926 |
|  | MR-PRESSO | -0.65 (-0.90, -0.40) | 0.001 | 18.595 | 0.148 |  |  |
| Free cholesterol in very large VLDL | IVW | 0.18 (-0.01, 0.38) | 0.070 | 0.947 | 1.000 |  |  |
|  | MR-Egger | 0.41 (-0.87, 1.68) | 0.551 | 0.828 | 0.999 | 0.003 | 0.739 |
|  | MR-PRESSO | 0.18 (0.12, 0.25) | 3.39e-04 | 1.206 | 1.000 |  |  |
| Free cholesterol in very small VLDL | IVW | -0.04 (-0.24, 0.16) | 0.712 | 1.506 | 0.997 |  |  |
|  | MR-Egger | -0.51 (-1.81, 0.78) | 0.461 | 0.980 | 0.998 | -0.007 | 0.489 |
|  | MR-PRESSO | -0.04 (-0.12, 0.04) | 0.390 | 1.913 | 0.998 |  |  |
| **Glycolysis** |  |  |  |  |  |  |  |
| Citrate | IVW | -0.33 (-0.54, -0.13) | 0.001 | 5.924 | 0.748 |  |  |
|  | MR-Egger | -1.07 (-2.38, 0.24) | 0.149 | 4.688 | 0.790 | -0.010 | 0.299 |
|  | MR-PRESSO | -0.33 (-0.50, -0.17) | 0.003 | 7.373 | 0.769 |  |  |
| Glucose | IVW | 0.20 (0, 0.41) | 0.054 | 3.044 | 0.963 |  |  |
|  | MR-Egger | 0.54 (-0.78, 1.87) | 0.444 | 2.782 | 0.947 | 0.005 | 0.623 |
|  | MR-PRESSO | 0.20 (0.08, 0.32) | 0.009 | 3.651 | 0.968 |  |  |
| Lactate | IVW | 0 (-0.23, 0.24) | 0.979 | 11.792 | 0.225 |  |  |
|  | MR-Egger | 0.2 (-1.39, 1.80) | 0.810 | 11.702 | 0.165 | 0.003 | 0.810 |
|  | MR-PRESSO | 0 (-0.23, 0.24) | 0.980 | 14.539 | 0.232 |  |  |
| **Ketone bodies** |  |  |  |  |  |  |  |
| 3-Hydroxybutyrate | IVW | -0.32 (-0.57, -0.07) | 0.012 | 13.686 | 0.134 |  |  |
|  | MR-Egger | -0.99 (-2.65, 0.66) | 0.273 | 12.657 | 0.124 | -0.009 | 0.443 |
|  | MR-PRESSO | -0.32 (-0.57, -0.07) | 0.034 | 16.899 | 0.175 |  |  |
| Acetate | IVW | -0.17 (-0.38, 0.03) | 0.097 | 6.024 | 0.738 |  |  |
|  | MR-Egger | -0.16 (-1.49, 1.17) | 0.821 | 6.023 | 0.645 | 0.000 | 0.982 |
|  | MR-PRESSO | -0.17 (-0.34, -0.01) | 0.073 | 7.326 | 0.760 |  |  |
| Acetoacetate | IVW | 0.02 (-0.18, 0.22) | 0.846 | 7.535 | 0.582 |  |  |
|  | MR-Egger | -0.37 (-1.69, 0.95) | 0.597 | 7.189 | 0.516 | -0.005 | 0.573 |
|  | MR-PRESSO | 0.02 (-0.17, 0.21) | 0.837 | 9.264 | 0.593 |  |  |
| Acetone | IVW | -0.45 (-0.67, -0.23) | 5.55e-05 | 10.234 | 0.332 |  |  |
|  | MR-Egger | -0.82 (-2.29, 0.65) | 0.305 | 9.920 | 0.271 | -0.005 | 0.629 |
|  | MR-PRESSO | -0.45 (-0.67, -0.23) | 0.003 | 12.476 | 0.386 |  |  |
| Albumin | IVW | -0.09 (-0.29, 0.12) | 0.397 | 7.467 | 0.589 |  |  |
|  | MR-Egger | -0.85 (-2.17, 0.47) | 0.242 | 6.157 | 0.630 | -0.010 | 0.285 |
|  | MR-PRESSO | -0.09 (-0.28, 0.10) | 0.377 | 10.004 | 0.534 |  |  |
| Creatinine | IVW | 0.14 (-0.04, 0.31) | 0.130 | 5.783 | 0.761 |  |  |
|  | MR-Egger | -0.84 (-1.98, 0.29) | 0.184 | 2.865 | 0.943 | -0.013 | 0.126 |
|  | MR-PRESSO | 0.14 (-0.01, 0.28) | 0.091 | 7.506 | 0.748 |  |  |
| Glycoprotein acetyls | IVW | 0.29 (0.09, 0.49) | 0.005 | 2.295 | 0.986 |  |  |
|  | MR-Egger | -0.36 (-1.67, 0.95) | 0.604 | 1.321 | 0.995 | -0.009 | 0.353 |
|  | MR-PRESSO | 0.29 (0.19, 0.39) | 3.61e-04 | 2.867 | 0.983 |  |  |
| Pyruvate | IVW | -0.49 (-0.69, -0.28) | 3.26e-06 | 2.955 | 0.966 |  |  |
|  | MR-Egger | -0.02 (-1.34, 1.30) | 0.981 | 2.457 | 0.964 | 0.006 | 0.500 |
|  | MR-PRESSO | -0.49 (-0.60, -0.37) | 1.96e-05 | 3.853 | 0.974 |  |  |
| **Lipoprotein particles** |  |  |  |  |  |  |  |
| Concentration of HDL particles | IVW | 0.43 (0.23, 0.62) | 1.37e-05 | 6.571 | 0.682 |  |  |
|  | MR-Egger | -0.81 (-2.05, 0.43) | 0.236 | 2.649 | 0.954 | -0.017 | 0.083 |
|  | MR-PRESSO | 0.43 (0.26, 0.59) | 0.001 | 8.912 | 0.665 |  |  |
| Concentration of IDL particles | IVW | -0.04 (-0.24, 0.17) | 0.734 | 3.898 | 0.918 |  |  |
|  | MR-Egger | -0.98 (-2.29, 0.32) | 0.178 | 1.816 | 0.986 | -0.013 | 0.187 |
|  | MR-PRESSO | -0.04 (-0.17, 0.10) | 0.619 | 5.053 | 0.920 |  |  |
| Concentration of LDL particles | IVW | 0.12 (-0.08, 0.33) | 0.244 | 2.659 | 0.976 |  |  |
|  | MR-Egger | -0.70 (-2.02, 0.62) | 0.328 | 1.126 | 0.997 | -0.011 | 0.251 |
|  | MR-PRESSO | 0.12 (0.01, 0.23) | 0.061 | 3.406 | 0.970 |  |  |
| Concentration of VLDL particles | IVW | 0.16 (-0.04, 0.36) | 0.126 | 0.403 | 1.000 |  |  |
|  | MR-Egger | -0.12 (-1.44, 1.19) | 0.857 | 0.219 | 1.000 | -0.004 | 0.680 |
|  | MR-PRESSO | 0.16 (0.12, 0.20) | 4.87e-05 | 0.483 | 1.000 |  |  |
| Concentration of chylomicrons and extremely large VLDL particles | IVW | 0.16 (-0.04, 0.35) | 0.119 | 1.512 | 0.997 |  |  |
|  | MR-Egger | 0.59 (-0.68, 1.86) | 0.386 | 1.044 | 0.998 | 0.006 | 0.513 |
|  | MR-PRESSO | 0.16 (0.08, 0.24) | 0.004 | 1.934 | 0.995 |  |  |
| Concentration of large HDL particles | IVW | -0.19 (-0.39, 0) | 0.055 | 11.018 | 0.274 |  |  |
|  | MR-Egger | -0.63 (-1.94, 0.69) | 0.378 | 10.461 | 0.234 | -0.006 | 0.532 |
|  | MR-PRESSO | -0.19 (-0.39, 0) | 0.087 | 13.619 | 0.310 |  |  |
| Concentration of large LDL particles | IVW | 0.16 (-0.04, 0.37) | 0.122 | 3.233 | 0.954 |  |  |
|  | MR-Egger | -0.77 (-2.09, 0.54) | 0.283 | 1.253 | 0.996 | -0.013 | 0.197 |
|  | MR-PRESSO | 0.16 (0.04, 0.28) | 0.030 | 4.163 | 0.951 |  |  |
| Concentration of large VLDL particles | IVW | 0.26 (0.06, 0.46) | 0.011 | 1.351 | 0.998 |  |  |
|  | MR-Egger | 0.42 (-0.85, 1.70) | 0.534 | 1.285 | 0.996 | 0.002 | 0.804 |
|  | MR-PRESSO | 0.26 (0.18, 0.33) | 1.02e-04 | 1.673 | 0.998 |  |  |
| Concentration of medium HDL particles | IVW | 0.31 (0.12, 0.50) | 0.001 | 5.030 | 0.832 |  |  |
|  | MR-Egger | -0.57 (-1.79, 0.64) | 0.381 | 2.940 | 0.938 | -0.012 | 0.186 |
|  | MR-PRESSO | 0.31 (0.17, 0.45) | 0.002 | 6.750 | 0.814 |  |  |
| Concentration of medium LDL particles | IVW | 0.06 (-0.14, 0.27) | 0.541 | 2.046 | 0.991 |  |  |
|  | MR-Egger | -0.58 (-1.90, 0.74) | 0.415 | 1.111 | 0.997 | -0.009 | 0.362 |
|  | MR-PRESSO | 0.06 (-0.03, 0.16) | 0.231 | 2.567 | 0.994 |  |  |
| Concentration of medium VLDL particles | IVW | 0.15 (-0.06, 0.35) | 0.156 | 0.781 | 1.000 |  |  |
|  | MR-Egger | -0.32 (-1.64, 1) | 0.646 | 0.283 | 1.000 | -0.006 | 0.500 |
|  | MR-PRESSO | 0.15 (0.09, 0.21) | 0.001 | 0.966 | 1.000 |  |  |
| Concentration of small HDL particles | IVW | 0.64 (0.43, 0.84) | 9.44e-10 | 6.092 | 0.731 |  |  |
|  | MR-Egger | -0.67 (-1.99, 0.65) | 0.346 | 2.192 | 0.975 | -0.018 | 0.084 |
|  | MR-PRESSO | 0.64 (0.47, 0.81) | 3.95e-05 | 7.848 | 0.790 |  |  |
| Concentration of small LDL particles | IVW | 0.04 (-0.16, 0.25) | 0.679 | 1.550 | 0.997 |  |  |
|  | MR-Egger | -0.52 (-1.84, 0.80) | 0.464 | 0.840 | 0.999 | -0.008 | 0.424 |
|  | MR-PRESSO | 0.04 (-0.04, 0.13) | 0.345 | 1.954 | 0.997 |  |  |
| Concentration of small VLDL particles | IVW | 0.25 (0.05, 0.45) | 0.016 | 0.903 | 1.000 |  |  |
|  | MR-Egger | 0.05 (-1.25, 1.36) | 0.937 | 0.817 | 0.999 | -0.003 | 0.776 |
|  | MR-PRESSO | 0.25 (0.18, 0.31) | 3.40e-05 | 1.067 | 1.000 |  |  |
| Concentration of very large HDL particles | IVW | -0.49 (-0.73, -0.25) | 6.35e-05 | 15.469 | 0.079 |  |  |
|  | MR-Egger | -0.60 (-2.24, 1.05) | 0.495 | 15.436 | 0.051 | -0.001 | 0.900 |
|  | MR-PRESSO | -0.49 (-0.73, -0.25) | 0.003 | 18.655 | 0.144 |  |  |
| Concentration of very large VLDL particles | IVW | 0.25 (0.06, 0.45) | 0.011 | 1.759 | 0.995 |  |  |
|  | MR-Egger | 0.53 (-0.74, 1.80) | 0.435 | 1.571 | 0.991 | 0.004 | 0.676 |
|  | MR-PRESSO | 0.25 (0.17, 0.34) | 2.84e-04 | 2.215 | 0.996 |  |  |
| Concentration of very small VLDL particles | IVW | 0.02 (-0.18, 0.22) | 0.844 | 1.185 | 0.999 |  |  |
|  | MR-Egger | -0.48 (-1.78, 0.82) | 0.491 | 0.604 | 1.000 | -0.007 | 0.468 |
|  | MR-PRESSO | 0.02 (-0.05, 0.09) | 0.602 | 1.514 | 0.999 |  |  |
| **Phospholipids** |  |  |  |  |  |  |  |
| Phospholipids in HDL | IVW | 0.17 (-0.01, 0.36) | 0.066 | 5.232 | 0.814 |  |  |
|  | MR-Egger | -0.45 (-1.64, 0.74) | 0.480 | 4.151 | 0.843 | -0.009 | 0.329 |
|  | MR-PRESSO | 0.17 (0.03, 0.31) | 0.039 | 6.794 | 0.798 |  |  |
| Phospholipids in IDL | IVW | 0.06 (-0.14, 0.26) | 0.562 | 5.886 | 0.751 |  |  |
|  | MR-Egger | -0.96 (-2.24, 0.31) | 0.176 | 3.347 | 0.911 | -0.014 | 0.150 |
|  | MR-PRESSO | 0.06 (-0.10, 0.22) | 0.492 | 7.633 | 0.744 |  |  |
| Phospholipids in LDL | IVW | 0.21 (0, 0.41) | 0.048 | 4.420 | 0.882 |  |  |
|  | MR-Egger | -0.91 (-2.23, 0.41) | 0.212 | 1.584 | 0.991 | -0.015 | 0.131 |
|  | MR-PRESSO | 0.21 (0.06, 0.35) | 0.020 | 5.766 | 0.866 |  |  |
| Phospholipids in VLDL | IVW | 0.19 (-0.01, 0.39) | 0.069 | 0.362 | 1.000 |  |  |
|  | MR-Egger | 0.06 (-1.25, 1.36) | 0.934 | 0.323 | 1.000 | -0.002 | 0.848 |
|  | MR-PRESSO | 0.19 (0.15, 0.23) | 8.15e-06 | 0.433 | 1.000 |  |  |
| Phospholipids in chylomicrons and extremely large VLDL | IVW | 0.16 (-0.04, 0.35) | 0.117 | 1.983 | 0.992 |  |  |
|  | MR-Egger | 0.64 (-0.62, 1.91) | 0.349 | 1.405 | 0.994 | 0.007 | 0.469 |
|  | MR-PRESSO | 0.16 (0.07, 0.25) | 0.009 | 2.539 | 0.994 |  |  |
| Phospholipids in large HDL | IVW | -0.15 (-0.33, 0.03) | 0.112 | 8.639 | 0.471 |  |  |
|  | MR-Egger | -0.49 (-1.67, 0.68) | 0.434 | 8.281 | 0.406 | -0.005 | 0.573 |
|  | MR-PRESSO | -0.15 (-0.32, 0.03) | 0.139 | 10.664 | 0.496 |  |  |
| Phospholipids in large LDL | IVW | 0.19 (-0.01, 0.40) | 0.062 | 5.431 | 0.795 |  |  |
|  | MR-Egger | -1.02 (-2.33, 0.29) | 0.165 | 2.041 | 0.980 | -0.017 | 0.103 |
|  | MR-PRESSO | 0.19 (0.04, 0.35) | 0.040 | 7.113 | 0.770 |  |  |
| Phospholipids in large VLDL | IVW | 0.25 (0.05, 0.45) | 0.013 | 1.890 | 0.993 |  |  |
|  | MR-Egger | 0.50 (-0.78, 1.77) | 0.466 | 1.743 | 0.988 | 0.003 | 0.711 |
|  | MR-PRESSO | 0.25 (0.16, 0.34) | 4.11e-04 | 2.369 | 0.992 |  |  |
| Phospholipids in medium HDL | IVW | 0.36 (0.17, 0.55) | 2.37e-04 | 3.216 | 0.955 |  |  |
|  | MR-Egger | -0.34 (-1.57, 0.90) | 0.608 | 1.968 | 0.982 | -0.010 | 0.296 |
|  | MR-PRESSO | 0.36 (0.24, 0.47) | 1.69e-04 | 4.333 | 0.945 |  |  |
| Phospholipids in medium LDL | IVW | 0.28 (0.07, 0.48) | 0.008 | 3.517 | 0.940 |  |  |
|  | MR-Egger | -0.76 (-2.08, 0.56) | 0.293 | 1.096 | 0.998 | -0.014 | 0.158 |
|  | MR-PRESSO | 0.28 (0.15, 0.41) | 0.002 | 4.530 | 0.949 |  |  |
| Phospholipids in medium VLDL | IVW | 0.15 (-0.05, 0.36) | 0.145 | 0.907 | 1.000 |  |  |
|  | MR-Egger | -0.36 (-1.68, 0.96) | 0.607 | 0.314 | 1.000 | -0.007 | 0.463 |
|  | MR-PRESSO | 0.15 (0.09, 0.22) | 0.001 | 1.122 | 0.999 |  |  |
| Phospholipids in small HDL | IVW | 0.61 (0.41, 0.81) | 4.38e-09 | 3.994 | 0.912 |  |  |
|  | MR-Egger | -0.25 (-1.56, 1.06) | 0.717 | 2.297 | 0.971 | -0.012 | 0.229 |
|  | MR-PRESSO | 0.61 (0.47, 0.74) | 1.02e-05 | 5.105 | 0.930 |  |  |
| Phospholipids in small LDL | IVW | 0.07 (-0.13, 0.28) | 0.478 | 2.716 | 0.974 |  |  |
|  | MR-Egger | -0.65 (-1.97, 0.67) | 0.361 | 1.525 | 0.992 | -0.010 | 0.307 |
|  | MR-PRESSO | 0.07 (-0.04, 0.19) | 0.229 | 3.469 | 0.973 |  |  |
| Phospholipids in small VLDL | IVW | 0.20 (0, 0.41) | 0.050 | 1.140 | 0.999 |  |  |
|  | MR-Egger | -0.37 (-1.68, 0.95) | 0.600 | 0.399 | 1.000 | -0.008 | 0.414 |
|  | MR-PRESSO | 0.20 (0.13, 0.28) | 3.71e-04 | 1.394 | 1.000 |  |  |
| Phospholipids in very large HDL | IVW | -0.54 (-0.77, -0.32) | 1.61e-06 | 13.155 | 0.156 |  |  |
|  | MR-Egger | -0.48 (-1.99, 1.04) | 0.555 | 13.142 | 0.107 | 0.001 | 0.933 |
|  | MR-PRESSO | -0.54 (-0.77, -0.32) | 0.001 | 15.771 | 0.248 |  |  |
| Phospholipids in very large VLDL | IVW | 0.21 (0.01, 0.41) | 0.036 | 1.061 | 0.999 |  |  |
|  | MR-Egger | 0.44 (-0.83, 1.72) | 0.516 | 0.933 | 0.999 | 0.003 | 0.730 |
|  | MR-PRESSO | 0.21 (0.14, 0.28) | 1.75e-04 | 1.344 | 1.000 |  |  |
| Phospholipids in very small VLDL | IVW | 0 (-0.20, 0.20) | 0.979 | 0.483 | 1.000 |  |  |
|  | MR-Egger | -0.25 (-1.56, 1.05) | 0.711 | 0.328 | 1.000 | -0.004 | 0.705 |
|  | MR-PRESSO | 0 (-0.04, 0.05) | 0.912 | 0.594 | 1.000 |  |  |
| **Lipoprotein particles Size&Apolipoproteins** |  |  |  |  |  |  |  |
| Apolipoprotein A1 | IVW | 0.28 (0.09, 0.47) | 0.004 | 5.996 | 0.740 |  |  |
|  | MR-Egger | -0.63 (-1.83, 0.58) | 0.339 | 3.779 | 0.876 | -0.012 | 0.175 |
|  | MR-PRESSO | 0.28 (0.13, 0.43) | 0.006 | 7.975 | 0.719 |  |  |
| Apolipoprotein B | IVW | 0.10 (-0.11, 0.30) | 0.352 | 2.517 | 0.980 |  |  |
|  | MR-Egger | -0.71 (-2.03, 0.61) | 0.324 | 1.050 | 0.998 | -0.011 | 0.260 |
|  | MR-PRESSO | 0.10 (-0.01, 0.21) | 0.112 | 3.229 | 0.981 |  |  |
| Average diameter for HDL particles | IVW | -0.32 (-0.52, -0.13) | 0.001 | 10.713 | 0.296 |  |  |
|  | MR-Egger | -0.35 (-1.70, 1.00) | 0.624 | 10.711 | 0.219 | 0.000 | 0.968 |
|  | MR-PRESSO | -0.32 (-0.52, -0.13) | 0.011 | 12.879 | 0.352 |  |  |
| Average diameter for LDL particles | IVW | 0.30 (0.10, 0.50) | 0.003 | 4.177 | 0.899 |  |  |
|  | MR-Egger | -0.71 (-2.00, 0.58) | 0.314 | 1.759 | 0.988 | -0.014 | 0.159 |
|  | MR-PRESSO | 0.30 (0.17, 0.44) | 0.002 | 5.405 | 0.903 |  |  |
| Average diameter for VLDL particles | IVW | 0.28 (0.09, 0.47) | 0.004 | 1.625 | 0.996 |  |  |
|  | MR-Egger | 0.47 (-0.76, 1.70) | 0.473 | 1.526 | 0.992 | 0.003 | 0.761 |
|  | MR-PRESSO | 0.28 (0.20, 0.36) | 8.94e-05 | 2.009 | 0.997 |  |  |
| **Total lipids** |  |  |  |  |  |  |  |
| Total lipids in HDL | IVW | 0.15 (-0.03, 0.34) | 0.102 | 6.666 | 0.672 |  |  |
|  | MR-Egger | -0.57 (-1.76, 0.61) | 0.369 | 5.173 | 0.739 | -0.010 | 0.257 |
|  | MR-PRESSO | 0.15 (0, 0.31) | 0.089 | 8.648 | 0.659 |  |  |
| Total lipids in IDL | IVW | 0.07 (-0.12, 0.27) | 0.461 | 5.692 | 0.770 |  |  |
|  | MR-Egger | -0.99 (-2.26, 0.29) | 0.168 | 2.972 | 0.936 | -0.015 | 0.138 |
|  | MR-PRESSO | 0.07 (-0.08, 0.23) | 0.378 | 7.453 | 0.737 |  |  |
| Total lipids in LDL | IVW | 0.24 (0.03, 0.44) | 0.022 | 4.623 | 0.866 |  |  |
|  | MR-Egger | -0.89 (-2.21, 0.42) | 0.220 | 1.717 | 0.988 | -0.016 | 0.127 |
|  | MR-PRESSO | 0.24 (0.09, 0.38) | 0.011 | 6.052 | 0.858 |  |  |
| Total lipids in VLDL | IVW | 0.23 (0.03, 0.43) | 0.026 | 0.427 | 1.000 |  |  |
|  | MR-Egger | 0.19 (-1.10, 1.49) | 0.780 | 0.424 | 1.000 | -0.001 | 0.956 |
|  | MR-PRESSO | 0.23 (0.18, 0.27) | 3.06e-06 | 0.521 | 1.000 |  |  |
| Total lipids in chylomicrons and extremely large VLDL | IVW | 0.13 (-0.07, 0.33) | 0.189 | 1.241 | 0.999 |  |  |
|  | MR-Egger | 0.59 (-0.68, 1.86) | 0.390 | 0.731 | 0.999 | 0.006 | 0.495 |
|  | MR-PRESSO | 0.13 (0.06, 0.21) | 0.006 | 1.591 | 0.999 |  |  |
| Total lipids in large HDL | IVW | -0.18 (-0.37, 0.01) | 0.062 | 9.856 | 0.362 |  |  |
|  | MR-Egger | -0.57 (-1.82, 0.68) | 0.396 | 9.397 | 0.310 | -0.005 | 0.549 |
|  | MR-PRESSO | -0.18 (-0.37, 0.01) | 0.095 | 12.181 | 0.380 |  |  |
| Total lipids in large LDL | IVW | 0.24 (0.04, 0.44) | 0.021 | 5.833 | 0.757 |  |  |
|  | MR-Egger | -0.99 (-2.29, 0.32) | 0.177 | 2.368 | 0.968 | -0.017 | 0.100 |
|  | MR-PRESSO | 0.24 (0.08, 0.40) | 0.018 | 7.652 | 0.734 |  |  |
| Total lipids in large VLDL | IVW | 0.27 (0.07, 0.47) | 0.007 | 1.273 | 0.999 |  |  |
|  | MR-Egger | 0.42 (-0.85, 1.70) | 0.532 | 1.215 | 0.996 | 0.002 | 0.817 |
|  | MR-PRESSO | 0.27 (0.20, 0.35) | 5.47e-05 | 1.576 | 1.000 |  |  |
| Total lipids in lipoprotein particles | IVW | 0.25 (0.05, 0.45) | 0.015 | 3.128 | 0.959 |  |  |
|  | MR-Egger | -0.57 (-1.87, 0.72) | 0.412 | 1.542 | 0.992 | -0.011 | 0.243 |
|  | MR-PRESSO | 0.25 (0.13, 0.37) | 0.003 | 4.151 | 0.948 |  |  |
| Total lipids in medium HDL | IVW | 0.35 (0.16, 0.54) | 3.33e-04 | 4.062 | 0.907 |  |  |
|  | MR-Egger | -0.47 (-1.69, 0.75) | 0.472 | 2.300 | 0.970 | -0.011 | 0.221 |
|  | MR-PRESSO | 0.35 (0.22, 0.48) | 4.68e-04 | 5.483 | 0.900 |  |  |
| Total lipids in medium LDL | IVW | 0.26 (0.05, 0.46) | 0.015 | 3.070 | 0.961 |  |  |
|  | MR-Egger | -0.71 (-2.03, 0.61) | 0.323 | 0.974 | 0.998 | -0.013 | 0.186 |
|  | MR-PRESSO | 0.26 (0.14, 0.37) | 0.002 | 3.970 | 0.959 |  |  |
| Total lipids in medium VLDL | IVW | 0.21 (0.01, 0.42) | 0.039 | 0.575 | 1.000 |  |  |
|  | MR-Egger | -0.15 (-1.46, 1.16) | 0.824 | 0.263 | 1.000 | -0.005 | 0.592 |
|  | MR-PRESSO | 0.21 (0.16, 0.27) | 1.84e-05 | 0.692 | 1.000 |  |  |
| Total lipids in small HDL | IVW | 0.65 (0.44, 0.85) | 5.12e-10 | 4.769 | 0.854 |  |  |
|  | MR-Egger | -0.37 (-1.68, 0.95) | 0.597 | 2.418 | 0.965 | -0.014 | 0.164 |
|  | MR-PRESSO | 0.65 (0.50, 0.80) | 1.31e-05 | 6.086 | 0.887 |  |  |
| Total lipids in small LDL | IVW | 0.15 (-0.05, 0.36) | 0.149 | 2.505 | 0.981 |  |  |
|  | MR-Egger | -0.67 (-1.99, 0.65) | 0.352 | 0.998 | 0.998 | -0.011 | 0.254 |
|  | MR-PRESSO | 0.15 (0.04, 0.26) | 0.023 | 3.228 | 0.979 |  |  |
| Total lipids in small VLDL | IVW | 0.26 (0.05, 0.46) | 0.013 | 0.908 | 1.000 |  |  |
|  | MR-Egger | -0.03 (-1.34, 1.28) | 0.965 | 0.718 | 0.999 | -0.004 | 0.674 |
|  | MR-PRESSO | 0.26 (0.19, 0.32) | 2.69e-05 | 1.076 | 1.000 |  |  |
| Total lipids in very large HDL | IVW | -0.53 (-0.77, -0.30) | 5.68e-06 | 14.212 | 0.115 |  |  |
|  | MR-Egger | -0.55 (-2.13, 1.02) | 0.510 | 14.210 | 0.076 | 0.000 | 0.980 |
|  | MR-PRESSO | -0.53 (-0.77, -0.30) | 0.001 | 17.098 | 0.200 |  |  |
| Total lipids in very large VLDL | IVW | 0.24 (0.04, 0.44) | 0.017 | 1.383 | 0.998 |  |  |
|  | MR-Egger | 0.49 (-0.78, 1.76) | 0.474 | 1.235 | 0.996 | 0.003 | 0.710 |
|  | MR-PRESSO | 0.24 (0.16, 0.32) | 1.82e-04 | 1.749 | 0.998 |  |  |
| Total lipids in very small VLDL | IVW | 0.04 (-0.17, 0.24) | 0.732 | 0.857 | 1.000 |  |  |
|  | MR-Egger | -0.37 (-1.66, 0.93) | 0.595 | 0.480 | 1.000 | -0.006 | 0.556 |
|  | MR-PRESSO | 0.04 (-0.03, 0.10) | 0.295 | 1.095 | 1.000 |  |  |
| **Triglycerides** |  |  |  |  |  |  |  |
| Triglycerides in HDL | IVW | 0.27 (0.07, 0.47) | 0.008 | 1.335 | 0.998 |  |  |
|  | MR-Egger | 0.53 (-0.77, 1.84) | 0.444 | 1.175 | 0.997 | 0.004 | 0.700 |
|  | MR-PRESSO | 0.27 (0.19, 0.35) | 7.46e-05 | 1.683 | 1.000 |  |  |
| Triglycerides in IDL | IVW | 0.17 (-0.03, 0.37) | 0.105 | 1.024 | 0.999 |  |  |
|  | MR-Egger | 0.32 (-0.99, 1.62) | 0.648 | 0.973 | 0.998 | 0.002 | 0.827 |
|  | MR-PRESSO | 0.17 (0.10, 0.24) | 0.001 | 1.283 | 0.999 |  |  |
| Triglycerides in LDL | IVW | 0.21 (0.01, 0.42) | 0.038 | 0.643 | 1.000 |  |  |
|  | MR-Egger | 0.19 (-1.12, 1.50) | 0.783 | 0.641 | 1.000 | 0.000 | 0.971 |
|  | MR-PRESSO | 0.21 (0.16, 0.27) | 2.84e-05 | 0.797 | 1.000 |  |  |
| Triglycerides in VLDL | IVW | 0.30 (0.10, 0.50) | 0.003 | 1.255 | 0.999 |  |  |
|  | MR-Egger | 0.50 (-0.78, 1.77) | 0.467 | 1.161 | 0.997 | 0.003 | 0.767 |
|  | MR-PRESSO | 0.30 (0.23, 0.37) | 2.33e-05 | 1.561 | 0.999 |  |  |
| Triglycerides in chylomicrons and extremely large VLDL | IVW | 0.06 (-0.14, 0.26) | 0.564 | 1.579 | 0.997 |  |  |
|  | MR-Egger | 0.65 (-0.62, 1.93) | 0.344 | 0.717 | 0.999 | 0.008 | 0.380 |
|  | MR-PRESSO | 0.06 (-0.02, 0.14) | 0.202 | 2.010 | 0.996 |  |  |
| Triglycerides in large HDL | IVW | 0.02 (-0.18, 0.21) | 0.867 | 1.499 | 0.997 |  |  |
|  | MR-Egger | 0.22 (-1.05, 1.48) | 0.746 | 1.401 | 0.994 | 0.003 | 0.762 |
|  | MR-PRESSO | 0.02 (-0.06, 0.10) | 0.691 | 1.798 | 0.998 |  |  |
| Triglycerides in large LDL | IVW | 0.20 (0, 0.40) | 0.056 | 0.695 | 1.000 |  |  |
|  | MR-Egger | 0.17 (-1.14, 1.48) | 0.805 | 0.694 | 1.000 | 0.000 | 0.968 |
|  | MR-PRESSO | 0.20 (0.14, 0.25) | 7.22e-05 | 0.865 | 1.000 |  |  |
| Triglycerides in large VLDL | IVW | 0.31 (0.12, 0.51) | 0.002 | 1.423 | 0.998 |  |  |
|  | MR-Egger | 0.50 (-0.77, 1.77) | 0.463 | 1.339 | 0.995 | 0.003 | 0.779 |
|  | MR-PRESSO | 0.31 (0.24, 0.39) | 2.58e-05 | 1.757 | 0.998 |  |  |
| Triglycerides in medium HDL | IVW | 0.31 (0.11, 0.51) | 0.002 | 2.044 | 0.991 |  |  |
|  | MR-Egger | 0.56 (-0.75, 1.86) | 0.427 | 1.905 | 0.984 | 0.003 | 0.719 |
|  | MR-PRESSO | 0.31 (0.22, 0.41) | 1.33e-04 | 2.539 | 0.989 |  |  |
| Triglycerides in medium LDL | IVW | 0.24 (0.04, 0.44) | 0.020 | 0.597 | 1.000 |  |  |
|  | MR-Egger | 0.16 (-1.14, 1.46) | 0.817 | 0.582 | 1.000 | -0.001 | 0.906 |
|  | MR-PRESSO | 0.24 (0.19, 0.29) | 8.39e-06 | 0.734 | 1.000 |  |  |
| Triglycerides in medium VLDL | IVW | 0.33 (0.13, 0.53) | 0.001 | 1.232 | 0.999 |  |  |
|  | MR-Egger | 0.31 (-0.97, 1.60) | 0.644 | 1.231 | 0.996 | 0.000 | 0.980 |
|  | MR-PRESSO | 0.33 (0.26, 0.40) | 1.01e-05 | 1.474 | 1.000 |  |  |
| Triglycerides in small HDL | IVW | 0.38 (0.18, 0.58) | 1.74e-04 | 4.548 | 0.872 |  |  |
|  | MR-Egger | 0.63 (-0.65, 1.91) | 0.363 | 4.399 | 0.819 | 0.003 | 0.710 |
|  | MR-PRESSO | 0.38 (0.24, 0.52) | 0.001 | 5.593 | 0.882 |  |  |
| Triglycerides in small LDL | IVW | 0.26 (0.06, 0.46) | 0.012 | 0.479 | 1.000 |  |  |
|  | MR-Egger | 0.27 (-1.01, 1.56) | 0.688 | 0.478 | 1.000 | 0.000 | 0.979 |
|  | MR-PRESSO | 0.26 (0.21, 0.30) | 1.75e-06 | 0.594 | 1.000 |  |  |
| Triglycerides in small VLDL | IVW | 0.31 (0.12, 0.51) | 0.002 | 2.291 | 0.986 |  |  |
|  | MR-Egger | 0.53 (-0.76, 1.81) | 0.444 | 2.183 | 0.975 | 0.003 | 0.751 |
|  | MR-PRESSO | 0.31 (0.21, 0.41) | 1.72e-04 | 2.814 | 0.991 |  |  |
| Triglycerides in very large HDL | IVW | -0.01 (-0.21, 0.19) | 0.908 | 1.415 | 0.998 |  |  |
|  | MR-Egger | 0.22 (-1.08, 1.52) | 0.747 | 1.288 | 0.996 | 0.003 | 0.731 |
|  | MR-PRESSO | -0.01 (-0.09, 0.07) | 0.777 | 1.678 | 0.999 |  |  |
| Triglycerides in very large VLDL | IVW | 0.26 (0.06, 0.45) | 0.010 | 1.920 | 0.993 |  |  |
|  | MR-Egger | 0.60 (-0.66, 1.86) | 0.380 | 1.631 | 0.990 | 0.005 | 0.605 |
|  | MR-PRESSO | 0.26 (0.17, 0.35) | 3.53e-04 | 2.429 | 0.990 |  |  |
| Triglycerides in very small VLDL | IVW | 0.22 (0.01, 0.42) | 0.036 | 1.923 | 0.993 |  |  |
|  | MR-Egger | 0.48 (-0.82, 1.78) | 0.493 | 1.765 | 0.987 | 0.004 | 0.701 |
|  | MR-PRESSO | 0.22 (0.12, 0.31) | 0.001 | 2.396 | 0.995 |  |  |

| **Supplementary Table 4. MR estimates of the effect of circulating metabolites on atrial fibrillation** | | | | | | | | |
| --- | --- | --- | --- | --- | --- | --- | --- | --- |
| Circulating metabolites | Method | β (95% CI) | OR (95% CI) | *P* | Q statistic | *P* -heterogeneity | Egger intercept | *P* -intercept |
| **Cholesterol** |  |  |  |  |  |  |  |  |
| Cholesterol in small HDL | IVW | -0.03 (-0.12, 0.05) | 0.97 (0.89, 1.05) | 0.459 | 52.793 | 0.482 |  |  |
|  | MR-Egger | -0.04 (-0.20, 0.12) | 0.96 (0.82, 1.13) | 0.608 | 52.771 | 0.444 | 0.001 | 0.884 |
|  | MR-PRESSO | -0.03 (-0.12, 0.05) | 0.97 (0.89, 1.05) | 0.436 | 60.754 | 0.406 |  |  |
| Cholesterol in very large HDL | IVW | -0.01 (-0.07, 0.06) | 0.99 (0.93, 1.06) | 0.827 | 80.925 | 0.992 |  |  |
|  | MR-Egger | -0.07 (-0.18, 0.03) | 0.93 (0.84, 1.03) | 0.179 | 78.551 | 0.994 | 0.004 | 0.126 |
|  | MR-PRESSO | -0.02 (-0.07, 0.04) | 0.98 (0.93, 1.04) | 0.572 | 105.430 | 0.973 |  |  |
| **Cholines** |  |  |  |  |  |  |  |  |
| Phosphatidylcholines | IVW | -0.06 (-0.13, 0.01) | 0.94 (0.88, 1.01) | 0.094 | 44.338 | 0.964 |  |  |
|  | MR-Egger | -0.04 (-0.16, 0.08) | 0.96 (0.85, 1.08) | 0.533 | 44.154 | 0.958 | -0.001 | 0.670 |
|  | MR-PRESSO | -0.07 (-0.13, -0.01) | 0.93 (0.88, 0.99) | 0.020 | 62.227 | 0.896 |  |  |
| **Compounds** |  |  |  |  |  |  |  |  |
| Total concentration of lipoprotein particles | IVW | -0.13 (-0.21, -0.04) | 0.88 (0.81, 0.96) | 0.002 | 58.332 | 0.573 |  |  |
|  | MR-Egger | -0.06 (-0.22, 0.10) | 0.94 (0.80, 1.11) | 0.454 | 57.450 | 0.569 | -0.004 | 0.351 |
|  | MR-PRESSO | -0.14 (-0.22, -0.06) | 0.87 (0.80, 0.94) | 0.001 | 71.807 | 0.477 |  |  |
| **Esterified cholesterol** |  |  |  |  |  |  |  |  |
| Cholesteryl esters in small HDL | IVW | -0.01 (-0.09, 0.07) | 0.99 (0.91, 1.07) | 0.842 | 42.456 | 0.661 |  |  |
|  | MR-Egger | -0.03 (-0.17, 0.11) | 0.97 (0.84, 1.12) | 0.724 | 42.369 | 0.625 | 0.001 | 0.770 |
|  | MR-PRESSO | 0 (-0.08, 0.07) | 1.00 (0.92, 1.07) | 0.933 | 45.759 | 0.724 |  |  |
| Cholesteryl esters in very large HDL | IVW | -0.02 (-0.08, 0.05) | 0.98 (0.92, 1.05) | 0.601 | 84.162 | 0.984 |  |  |
|  | MR-Egger | -0.05 (-0.16, 0.05) | 0.95 (0.85, 1.05) | 0.336 | 83.496 | 0.983 | 0.002 | 0.416 |
|  | MR-PRESSO | -0.03 (-0.08, 0.03) | 0.97 (0.92, 1.03) | 0.354 | 110.585 | 0.937 |  |  |
| **Fatty Acids** |  |  |  |  |  |  |  |  |
| Monounsaturated fatty acids | IVW | -0.07 (-0.16, 0.01) | 0.93 (0.85, 1.01) | 0.080 | 70.105 | 0.638 |  |  |
|  | MR-Egger | -0.10 (-0.27, 0.06) | 0.90 (0.76, 1.06) | 0.230 | 69.961 | 0.611 | 0.001 | 0.705 |
|  | MR-PRESSO | -0.04 (-0.11, 0.02) | 0.96 (0.90, 1.02) | 0.207 | 86.778 | 0.577 |  |  |
| **Free cholesterol** |  |  |  |  |  |  |  |  |
| Free cholesterol in small HDL | IVW | -0.07 (-0.16, 0.02) | 0.93 (0.85, 1.02) | 0.117 | 58.703 | 0.243 |  |  |
|  | MR-Egger | -0.03 (-0.22, 0.15) | 0.97 (0.80, 1.16) | 0.738 | 58.446 | 0.221 | -0.002 | 0.638 |
|  | MR-PRESSO | -0.09 (-0.18, 0) | 0.92 (0.84, 1.00) | 0.066 | 70.764 | 0.162 |  |  |
| Free cholesterol in very large HDL | IVW | -0.03 (-0.09, 0.03) | 0.97 (0.91, 1.03) | 0.366 | 66.807 | 0.988 |  |  |
|  | MR-Egger | -0.06 (-0.15, 0.03) | 0.94 (0.86, 1.03) | 0.211 | 66.029 | 0.987 | 0.002 | 0.380 |
|  | MR-PRESSO | -0.03 (-0.08, 0.03) | 0.97 (0.92, 1.03) | 0.317 | 86.176 | 0.923 |  |  |
| **Ketone bodies** |  |  |  |  |  |  |  |  |
| Acetone | IVW | -0.11 (-0.42, 0.20) | 0.90 (0.66, 1.22) | 0.493 | 8.171 | 0.517 |  |  |
|  | MR-Egger | -0.43 (-1.26, 0.39) | 0.65 (0.28, 1.48) | 0.331 | 7.466 | 0.487 | 0.012 | 0.425 |
|  | MR-PRESSO | 0.05 (-0.18, 0.28) | 1.05 (0.84, 1.32) | 0.691 | 13.259 | 0.482 |  |  |
| Pyruvate | IVW | 0.12 (-0.02, 0.26) | 1.12 (0.98, 1.30) | 0.105 | 14.345 | 0.706 |  |  |
|  | MR-Egger | 0.22 (-0.05, 0.49) | 1.25 (0.95, 1.63) | 0.131 | 13.589 | 0.696 | -0.006 | 0.397 |
|  | MR-PRESSO | 0.14 (0.02, 0.26) | 1.15 (1.02, 1.30) | 0.030 | 21.212 | 0.690 |  |  |
| **Lipoprotein particles** |  |  |  |  |  |  |  |  |
| Concentration of HDL particles | IVW | -0.12 (-0.20, -0.04) | 0.88 (0.82, 0.96) | 0.002 | 65.320 | 0.603 |  |  |
|  | MR-Egger | -0.09 (-0.24, 0.06) | 0.91 (0.79, 1.06) | 0.240 | 65.077 | 0.578 | -0.002 | 0.623 |
|  | MR-PRESSO | -0.11 (-0.19, -0.04) | 0.89 (0.83, 0.96) | 0.004 | 85.618 | 0.332 |  |  |
| Concentration of small HDL particles | IVW | -0.02 (-0.11, 0.06) | 0.98 (0.90, 1.06) | 0.611 | 50.980 | 0.357 |  |  |
|  | MR-Egger | -0.04 (-0.20, 0.13) | 0.96 (0.82, 1.14) | 0.647 | 50.925 | 0.322 | 0.001 | 0.823 |
|  | MR-PRESSO | -0.03 (-0.11, 0.06) | 0.97 (0.90, 1.06) | 0.559 | 59.263 | 0.382 |  |  |
| Concentration of very large HDL particles | IVW | -0.03 (-0.09, 0.04) | 0.98 (0.91, 1.04) | 0.430 | 78.391 | 0.980 |  |  |
|  | MR-Egger | -0.06 (-0.15, 0.03) | 0.94 (0.86, 1.03) | 0.208 | 77.406 | 0.980 | 0.003 | 0.323 |
|  | MR-PRESSO | -0.03 (-0.09, 0.02) | 0.97 (0.91, 1.02) | 0.203 | 100.235 | 0.952 |  |  |
| **Phospholipids** |  |  |  |  |  |  |  |  |
| Phospholipids in medium HDL | IVW | -0.10 (-0.18, -0.03) | 0.90 (0.84, 0.97) | 0.005 | 62.968 | 0.838 |  |  |
|  | MR-Egger | -0.07 (-0.20, 0.07) | 0.94 (0.82, 1.07) | 0.332 | 62.563 | 0.826 | -0.002 | 0.526 |
|  | MR-PRESSO | -0.10 (-0.17, -0.03) | 0.91 (0.84, 0.97) | 0.005 | 86.838 | 0.478 |  |  |
| Phospholipids in small HDL | IVW | -0.03 (-0.11, 0.05) | 0.97 (0.90, 1.05) | 0.455 | 62.746 | 0.450 |  |  |
|  | MR-Egger | -0.03 (-0.18, 0.13) | 0.97 (0.84, 1.14) | 0.745 | 62.739 | 0.414 | 0.000 | 0.934 |
|  | MR-PRESSO | -0.02 (-0.10, 0.06) | 0.98 (0.90, 1.06) | 0.581 | 69.981 | 0.416 |  |  |
| Phospholipids in very large HDL | IVW | -0.02 (-0.08, 0.05) | 0.98 (0.92, 1.05) | 0.585 | 79.685 | 0.885 |  |  |
|  | MR-Egger | -0.08 (-0.18, 0.01) | 0.92 (0.84, 1.01) | 0.098 | 76.653 | 0.916 | 0.005 | 0.085 |
|  | MR-PRESSO | -0.03 (-0.08, 0.03) | 0.97 (0.92, 1.03) | 0.316 | 104.144 | 0.825 |  |  |
| **Total lipids** |  |  |  |  |  |  |  |  |
| Total lipids in small HDL | IVW | 0 (-0.09, 0.08) | 1.00 (0.91, 1.08) | 0.950 | 52.736 | 0.523 |  |  |
|  | MR-Egger | -0.05 (-0.20, 0.10) | 0.95 (0.82, 1.11) | 0.513 | 52.179 | 0.506 | 0.003 | 0.459 |
|  | MR-PRESSO | 0 (-0.09, 0.08) | 1.00 (0.91, 1.08) | 0.906 | 54.691 | 0.536 |  |  |
| Total lipids in very large HDL | IVW | -0.02 (-0.08, 0.04) | 0.98 (0.92, 1.04) | 0.510 | 78.695 | 0.974 |  |  |
|  | MR-Egger | -0.06 (-0.16, 0.03) | 0.94 (0.85, 1.03) | 0.206 | 77.466 | 0.976 | 0.003 | 0.270 |
|  | MR-PRESSO | -0.03 (-0.08, 0.02) | 0.97 (0.92, 1.02) | 0.268 | 100.496 | 0.931 |  |  |
| **Triglycerides** |  |  |  |  |  |  |  |  |
| Triglycerides in small HDL | IVW | -0.08 (-0.16, 0) | 0.92 (0.85, 1.00) | 0.046 | 66.696 | 0.714 |  |  |
|  | MR-Egger | -0.02 (-0.17, 0.14) | 0.98 (0.84, 1.15) | 0.837 | 65.793 | 0.713 | -0.004 | 0.345 |
|  | MR-PRESSO | -0.04 (-0.10, 0.02) | 0.96 (0.90, 1.02) | 0.185 | 97.316 | 0.510 |  |  |
